# RNAi phenotypes are influenced by the genetic background of the injected strain

**DOI:** 10.1186/1471-2164-14-5

**Published:** 2013-01-16

**Authors:** Peter Kitzmann, Jonas Schwirz, Christian Schmitt-Engel, Gregor Bucher

**Affiliations:** 1 Department of Developmental Biology, Blumenbach Institute of Zoology, Göttingen Center of Molecular Biology, Georg-August-University Göttingen, Göttingen, Germany

**Keywords:** RNAi, Genetic background, Importin, Tribolium, Evolution, Emerging model organism

## Abstract

**Background:**

RNA interference (RNAi) is a powerful tool to study gene function in organisms that are not amenable to classical forward genetics. Hence, together with the ease of comprehensively identifying genes by new generation sequencing, RNAi is expanding the scope of animal species and questions that can be addressed in terms of gene function. In the case of genetic mutants, the genetic background of the strains used is known to influence the phenotype while this has not been described for RNAi experiments.

**Results:**

Here we show in the red flour beetle *Tribolium castaneum* that RNAi against *Tc*-*importin α1* leads to different phenotypes depending on the injected strain. We rule out off target effects and show that sequence divergence does not account for this difference. By quantitatively comparing phenotypes elicited by RNAi knockdown of four different genes we show that there is no general difference in RNAi sensitivity between these strains. Finally, we show that in case of *Tc*-*importin α1* the difference depends on the maternal genotype.

**Conclusions:**

These results show that in RNAi experiments strain specific differences have to be considered and that a proper documentation of the injected strain is required. This is especially important for the increasing number of emerging model organisms that are being functionally investigated using RNAi. In addition, our work shows that RNAi is suitable to systematically identify the differences in the gene regulatory networks present in populations of the same species, which will allow novel insights into the evolution of animal diversity.

## Background

For a long time, the identification of gene functions has been based on classical forward genetic screens where mutants are randomly generated, e.g. by chemical or transposon mediated mutagenesis. The established mutant strains are then screened for phenotypes and subsequently the disrupted gene is identified and further analyzed [1-5]. Importantly, it has been observed that the phenotypes of *Drosophila* and mouse mutants can depend on the genetic background of different strains, e.g. [6-13]. The same has been found for *E*.*coli*, rice and *C*.*elegans *[14-16]. In yeast, the portion of genes that are essential in only one of two closely related strains has been estimated to be about 6% [17]. The unbiased forward genetic approach to identify gene functions has been very successful but it also limits the questions that can be addressed. First, saturating screens are only feasible in a very small number of model organisms [1-5,18,19]. Within insects, this is true only for the fruit fly *Drosophila melanogaster* while a few non-saturating screens have been performed in other insects including the red flour beetle *Tribolium castaneum *[20-22]. The limitation to highly developed model organisms at the same time limits the scope of biological questions that can be asked. A further restriction of forward genetics is that mutant strains need to be maintained over time, which represents a significant effort feasible only with the support of large scientific communities. Moreover, the genetic tools, which facilitate stock keeping (e.g. balancer chromosomes) are not available in most organisms and are tedious to construct.

The discovery of RNA interference (RNAi) in animals [23] has opened the possibility to study gene function in many more animals and has significantly contributed to a an expansion of biological questions that are studied in terms of gene function. In RNAi, double stranded RNA (dsRNA) within cells is processed by the highly conserved RNAi machinery including the Dicer protein, which cuts the long dsRNA into 21mers. These are loaded into the destruction complex (RISC complex), which is guided by the single stranded small interfering RNAs (siRNAs) to mRNAs with complementary sequence. The Argonaute protein as part of the RISC complex eventually cuts the mRNAs within the region of complementary, leading to the destruction of the mRNA and consequently to a reduction of the gene product [24-26]. RNAi is an anti-viral defense system, is required for the silencing of transposons [27] and highly related processes act in post-transcriptional gene regulation, the control of chromatin and RNA polymerase II transcription elongation activity [24,28].

The RNAi response of some organisms is systemic, i.e. dsRNA delivered into the body cavity is distributed throughout the organism and enters all cells. Hence, local injection leads to systemic gene silencing [24,26,29,30]. In some organisms like *C*. *elegans* and *Tribolium* the RNAi effect is transmitted even from injected parents to the offspring [24,29-32].

RNAi in the red flour beetle *Tribolium castaneum* is robust, systemic, splice variant specific and feasible at all developmental stages [31-35]. Moreover, it is able to phenocopy genetic Null alleles at least in some instances, e.g. in the case of *Tcdfd *[33], *Tcdistalless *[32,36]*Tckrüppel *[37] and *Tcknirps* (Bucher, unpublished). The strength of the RNAi response can be experimentally modulated by varying the concentration of injected dsRNA or by varying the time between injection of the mother and collection of the phenotypic offspring [32,34,38-40].

In an ongoing genome wide RNAi screen in *Tribolium* (iBeetle screen, unpublished), females of the *black* strain [41] were injected with dsRNA of the fragment iB_00198 and were subsequently mated to *pig19* males [42]. In the cuticle of offspring first instar larvae, specific labrum defects were observed with high frequency. The knocked-down gene product is an Importin α, which belongs to the *karyopherin* multi-gene family of nuclear import receptors [43]. In metazoans three classes of *importin α* genes exist: *importin α1*, *importin α2*, and *importin α3 *[44]. Importin α proteins are nuclear import adaptors, which bind cargos containing a classical nuclear localization signal (cNLS) sequence [45]. The Importin α-cargo heterodimer forms a trimeric complex with the actual importer Importin β, which enables the passage of the cargo through the nuclear pore complex [46]. The Importin α1 protein shows a tandem array of ten armadillo (ARM) repeats, where the ARM domains 1 to 4 (major site) and the domains 4 to 8 (minor site) are responsible for recognition and binding of specific cargoes [47,48]. All members of the *importin α* family function the same way and it has been shown that they act redundantly on many cargoes but there are also cargoes, which require a specific Importin α for their nuclear import [44,45,49-54]. In the yeast *S*. *cerevisiae*, estimated 57% of steady-state nuclear proteins use this import system [45]. Considering this, it was surprising that the knock-down of a gene which encodes such a widely required factor would lead to such a specific cuticle phenotype in *Tribolium*.

In this work, we quantitatively compare the RNAi phenotypes of *Tc*-*importin α1* in two *Tribolium* laboratory strains, *black* and *San Bernadino* (*SB*). Surprisingly, we find that RNAi knock-down leads to qualitatively different phenotypes depending on the strain. Further, we show that this is neither due to a general difference in RNAi sensitivity of these strains nor to nucleotide sequence divergence between them or differential embryonic expression. Instead, we find that the genotype of the injected female determines the RNAi phenotype of the offspring. These results show that the phenotypes generated in RNAi experiments can depend on the genotype of the used strain and we suggest that a proper documentation of the strain is an essential piece of information when publishing RNAi studies in any species.

## Results and discussion

### iB_00198 dsRNA targets *Tc*-*importin α1*

**Figure 1:**
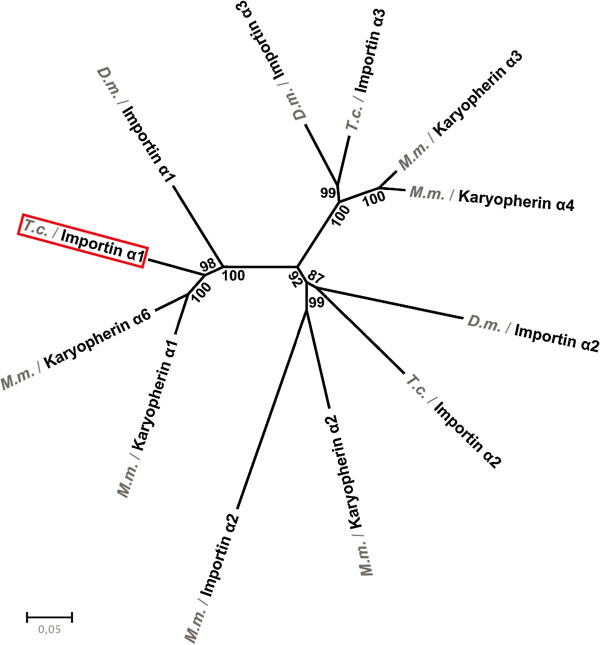
**Phylogenetic tree of Tc**-**Importin α proteins.** Metazoans have three conserved Importin α families (α1, α2 and α3). Tc-Importin α1 (framed) encodes a protein of the Importin α1 family. Interestingly, it is more closely related to the mouse than to the *Drosophila* orthologs. *M*. *musculus* has two paralogs per family while *Tribolium* and *Drosophila* have only one representative of each Importin α family. Shown is a Neighbor-joining tree with Bootstrap values for each node.

The RNAi phenotype of the iB_00198 dsRNA fragment revealed by the iBeetle screen was marked by a highly penetrant and specific loss of the labrum (The iBeetle consortium, unpublished). In order to follow up this phenotype, we first analyzed the phylogenetic relationship of the targeted gene. The iB_00198 sequence is part of the coding sequence of *TC000963* (Additional file 1 A). Phylogenetic analysis revealed that the *TC000963* gene is the single *importin α1* ortholog of *Tribolium*, called *Tc*-*importin α1* in the following (Figure 1, red frame; see Additional file 1 C for aligned sequences). The Tc-Importin α1 protein is more similar to the mouse orthologs (Karyopherin α1/6) than to *Drosophila* Importin α1. We checked the expression of *Tc*-*importin α1* by in situ hybridization and found it to be expressed ubiquitously in both *SB* and *black* embryos (0–26 h egg collection at 32°C; not shown).

### *Tc*-*importin α1* pRNAi cuticle phenotype is different in the two strains

**Figure 2:**
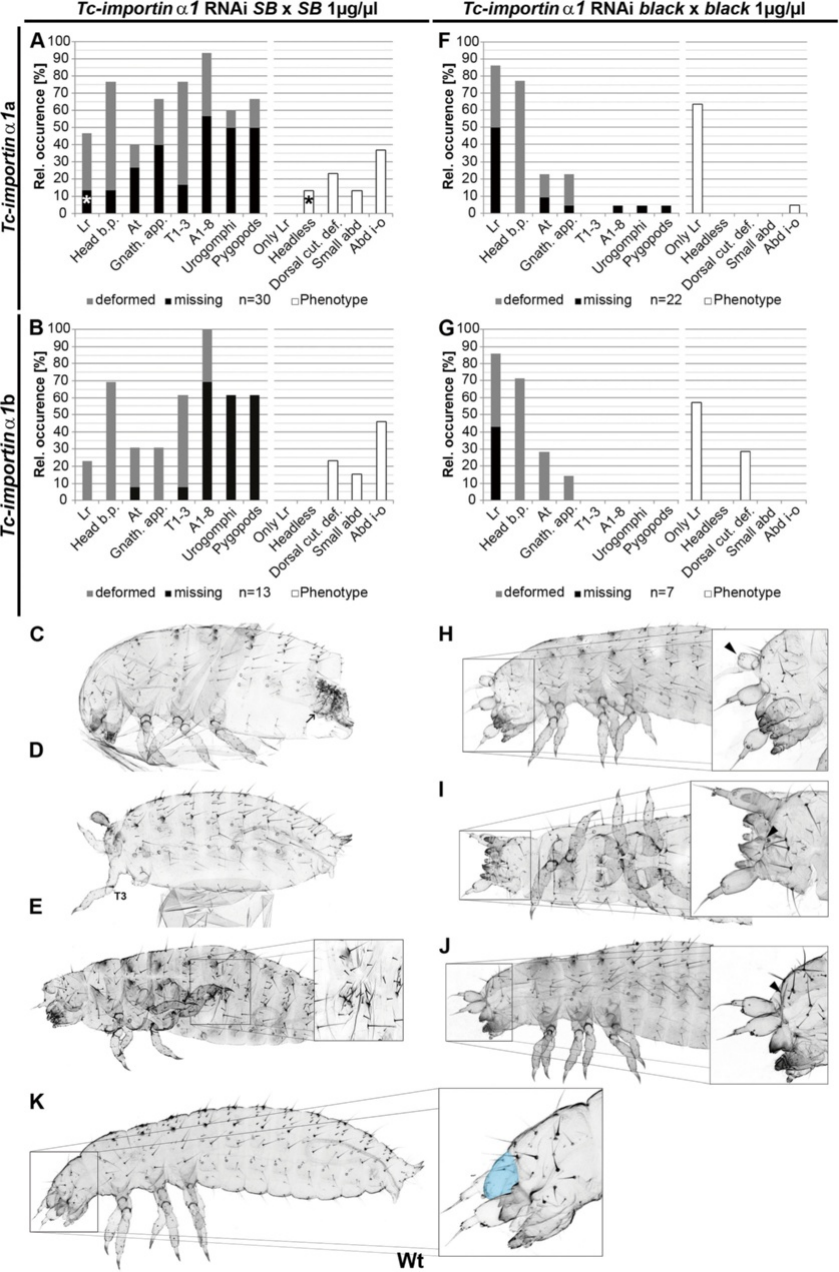
**Different *Tc***-***importin α1 *RNAi phenotypes in the *SB *and *black *strains.** (**A**, **B**, **F**, **G**) Quantification of affected cuticle structures (shaded bars, left part of the panels) and frequency of phenotype classes (white bars, right part of the panels). Note that these represent two separate analyses of the same set of RNAi cuticles and that not all cuticles could be assigned to a class. (**A**, **B**) Knock-down of *Tc*-*importin α1* in the *SB* strain using two non-overlapping fragments (*Tc*-*importin α1*a (**A**) or *Tc*-*importin α1*b (**B**)) results in a similar pleiotropic cuticle phenotype. Note that the loss of the labrum (white asterisk in A) is due to loss of the entire head (black asterisk) while a “labrum only” phenotype was not observed. (**C**) Lateral view of a cuticle showing the abdomen inside-out phenotype (“Abd i-o” class) where the posteriormost abdominal segments are involuted into the abdomen (black arrow). (**D**) Lateral view of a headless cuticle which lacks most anterior structures up to the third thoracic segment (T3). (E) Ventro-lateral view of a cuticle showing a mild dorsal cuticle defect. (**F**, **G**) The knock-down of *Tc*-*importin α1* in the *black* strain using the same non-overlapping fragments resulted in about 60% of the examined cuticles in a specific loss (**I**, **J**) or deformation (**H**) of the labrum (“Only Lr” class). (**H**) Lateral view of a cuticle showing a deformed labrum (black arrowhead). (**I**, **J**) Dorsal (**I**) and lateral (**J**) views of cuticles lacking the labrum (arrowhead). (K) Wild-type larval cuticle (Lr is marked in blue). *Lr* labrum, *Head b*.*p*. Head bristle pattern, *At* antennae, *Gnath*. *app*. gnathal appendages, *T1*-*3* thoracic segment 1–3, *A1*-*8* abdominal segments 1–8, *Dorsal cut*. *def*. dorsal cuticle defect, *Abd*. *i*-*o* abdomen inside-out.

**Figure 3:**
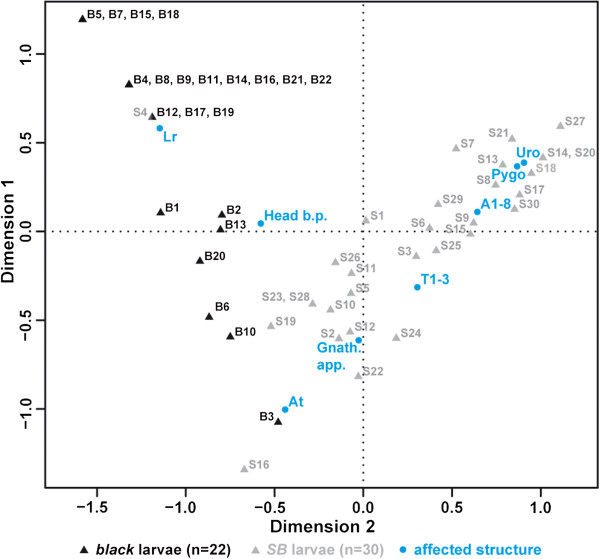
**Correspondence analysis of *Tc***-***importin α1 *a RNAi cuticle phenotypes in the *black *and *SB *background.** The individual *Tc*-*importin α1*a RNAi cuticles of the *black* genetic background (black triangles) group together at the left side of the plot (labrum, head bristles and antenna phenotypes) while the *SB* cuticle phenotypes (gray triangles) are located more on the right side of the plot (abdominal, thoracic and gnathal phenotypes). There is not much overlap between the two clusters.

To test whether the labrum specific iB_00198 phenotype detected under the high throughput conditions of the iBeetle screen was reproducible and not due to off target effects, two non-overlapping dsRNA fragments of the *Tc*-*importin α1* open reading frame were generated (Additional file 1 A and B) and injected into female pupae of the *SB* strain with the same dsRNA concentration as in the screen (1 μg/μl). The resulting cuticles were scored for deletion or malformation of different parts of the body (Figure 2A, B, indicated in black and gray, respectively). In addition, the number of individuals falling into one of several specific phenotype classes was determined (Figure 2A, white bars). Note that the “labrum only” class represents cuticles that show defects in the labrum but not in other structures. Some phenotypes did not fit any of the classes shown and were not similar enough to group them into an own class. Knock-down using the first fragment (*Tc*-*importin α1*a) resulted in a much stronger cuticle phenotype than observed in the screen. Most frequent were abdominal malformations (93.3%; n=30 cuticles), ranging from mild phenotypes in which the abdomen was constricted (“small abd”, 13.3%, not shown) to cuticles which showed an inversion of abdominal segments into the interior of the cuticle (abdomen inside-out: “Abd i-o”, 36.6%; Figure 2C). Defects in the thoracic segments were frequent (76.6%) where in most cases only single thoracic segments or legs were deformed (60%). Malformation of the head included mainly the loss (40%) or deformation (26.6%) of gnathal appendages (“Gnath. app.”). The antennae (“At”; 40%) and labrum (“Lr”; 46.6%) were also often affected after *Tc*-*importin α1* RNAi. In some cases, the complete head and adjacent thoracic segments were absent (“Headless”, 13.3%; Figure 2D). Dorsal cuticle defects (“Dorsal cut. def.”; Figure 2E) were found in 23.6% of examined larvae. Unexpectedly, the labrum-only defects observed in the iBeetle screen (i.e. cuticles showing no other defects apart from the labrum; “Only Lr”) were not observed in this experiment while overall, the defects were much more extensive. Of note, in all cases where the labrum was recorded as not being present (white asterisk in Figure 2A) this was due to complete loss of the head in the respective cuticle (black asterisk in Figure 2A). The results of the non-overlapping fragment (Tc-importin α1b, n=13) displayed quantitatively and qualitatively similar defects with somewhat reduced strength of labral and gnathal defects and the headless phenotype (Figure 2B). This made off target effects unlikely. In order to independently check the different quality of the phenotypes, we conducted a correspondence analysis of the data and visualized the results in a xy-plot. The phenotypes in the different strains form distinct clusters confirming the different quality of the phenotypes (Figure 3).

In order to test whether this unexpected phenotypic difference was due to the selection of dsRNA fragments different from the one used in the screen (Additional file 1A and B), or alternatively, from the use of a different strain, *Tc*-*importin α1* RNAi was repeated in the *black* strain. Both non-overlapping dsRNA fragments (1 μg/μl) were injected into *black* female pupae, which were mated with *black* males (Figure 2F-J) or *pig19* males (i.e. the combination used in the screen; Additional file 1 D). The knock-down using the Tc-importin α1a dsRNA (Figure 2F, n=22) frequently resulted in cuticles with an affected labrum (86.4%) which was either deformed (36.4%; Figure 2H) or completely absent (50%; Figure 2I, J). In a portion of the cuticles, other head defects (antennal and gnathal: 22.7%) or abdominal defects were found (4.5%). Notably, the “labrum only” phenotype was frequent (>60%). These observations were confirmed using the non-overlapping fragment with the only difference that additional dorsal cuticle defects were observed (28.6%; Figure 2G). To further confirm our finding, we repeated the RNAi with the original iB_000198 dsRNA fragment (1 μg/μl) in the *black* and *SB* strains, which resulted essentially in the same strain specific phenotypes (not shown; the original documentation of all *Tc*-*importin α1 *RNAi experiments is found in Additional file 1 E).

Taken together, these results showed that the knock-down of *Tcimportin α1* led to different phenotypes depending on which strain was injected and that this difference was not due to off target effects. Because both non-overlapping dsRNA fragments resulted in similar phenotypes, the following experiments were done using the Tc-importin α1a fragment. Importin α proteins are essential parts of the nuclear import machinery and have housekeeping functions [49]. Therefore, one would expect a dramatic and pleiotropic loss of function phenotype. The phenotype in the *SB* strain matches this expectation pretty well. Also in the *black* strain, some pleiotropic defects are observed, which increase somewhat in number at higher dsRNA concentrations. This is an indication that the expected pleiotropic phenotype is present but strongly reduced in the *black* strain.

#### The phenotypes are qualitatively different

**Figure 4:**
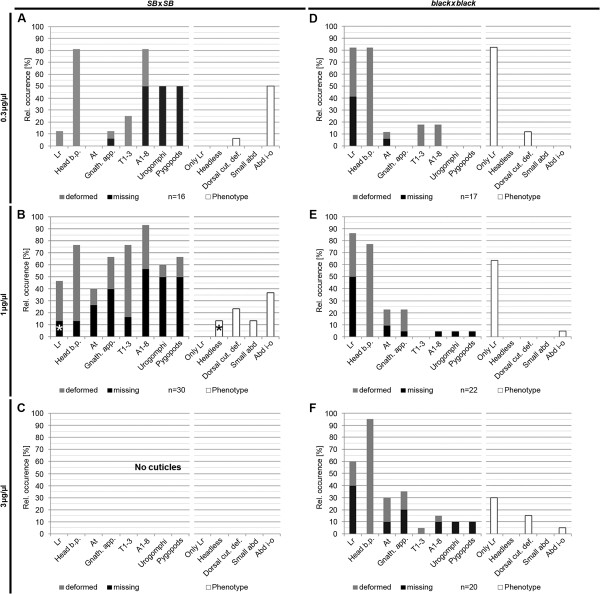
**Different phenotypic series.** Quantification of cuticle defects using different concentrations of dsRNA in the *SB* (**A-C**) and the *black* strains (**D-F**), panels B and E are taken from Figure 2. (**A**) Injection of 0.3 μg/μl dsRNA in the *SB* strain results in a similar phenotype as with 1 μg/μl (**B**) albeit with lower penetrance. (**C**) Knock-down in *SB* with 3 μg/μl dsRNA results in empty eggs (i.e. empty egg shells without cuticles in the cuticle preparation), which are indicator of severe defects which lead to the abortion of embryogenesis prior to cuticle formation. (**D**) RNAi in the *black* strain with 0.3 μg/μl leads to a similar phenotypic pattern as with 1 μg/μl (**E**). Besides a mild background of pleiotropic defects, loss or malformation of the labrum are the most frequent phenotype. Phenotypes, where the labrum is the only affected structure is represented by the bar labeled with “Only Lr”. (**F**) Injection of 3 μg/μl *Tc*-*importin α1* dsRNA leads to a similar portion of cuticles with an affected labrum accompanied with an increased portion of pleiotropic defects. As consequence, the fraction of cuticles that have labrum-only malformations drops to 30% although the labrum remains the most frequently affected structure.

RNAi experiments tend to produce a phenotypic series depending on the amount of injected dsRNA and due to experimental variation. Hence, despite the fact that the same concentration of dsRNA was injected in all experiments, the “labrum only” phenotype found in *black* could represent a weak phenotype while the more widespread defects found in *SB* could represent the stronger part of the phenotypic series. On the one hand, the additional defects that occurred in the *black* strain-albeit at low frequencies-could be interpreted in this way. On the other hand, the fact that the “labrum only” phenotype occurred very frequently in the *black* strain (Figure 2F,G) but never in the *SB* strain (Figure 2A,B) indicated a qualitative difference. If these different phenotypes were part of one phenotypic series, the injection of different amounts of dsRNAs in both strains was predicted to reveal concentrations where the phenotype pattern in both strains would overlap. Therefore, we injected both lower and higher concentrations of dsRNA into both strains (Figure 4).

Injecting lower amounts of dsRNA (0.3 μg/μl) into the *SB* strain led to a decreased frequency of cuticular defects, while their quality was similar to the 1 μg/μl experiment (compare Figure 4A with B). Specifically, the “labrum only” phenotype was not found. Using 3 μg/μl dsRNA led to an “empty egg” phenotype in all animals. Empty egg phenotypes are an indicator of very severe embryonic defects leading to the abortion of embryonic development prior to cuticle secretion resulting in empty egg shells in cuticle preparations (Figure 4C).

Knock-down of *Tc*-*importin α1* in the *black* strain using 0.3 μg/μl dsRNA resulted in a very similar phenotypic pattern as shown for 1 μg/μl (compare Figure 4D with E). Notably, “labrum only” phenotypes were found in about 80% (Figure 4D) where the labrum was absent in 41.2%. Other defects were observed with low frequency. Injection of 3 μg/μl dsRNA led to cuticles with a comparable occurrence of labrum defects but with a slightly increased portion of other cuticular defects (Figure 4E, F). As consequence, the number of “labrum only” (i.e. labrum but no other structure affected) phenotypes dropped to 30% but the labrum remained the most frequently deleted structure (40%). Finally, we tested the effect of *Tc*-*importin α1*RNAi in two other strains. We injected females of the *pig19* strain (derived from the *pearl* genetic background) and mated them with *black* males. The phenotype of the offspring was intermediate between *black* and *SB* injected females (Additional file 1I). In the *Georgia*-*2* (*GA*-*2*) genetic background, the injected females became sterile not allowing judging the cuticle phenotype of the offspring (Additional file 1 J).

In summary, the phenotypic series generated by RNAi with the same dsRNA in different strains differed qualitatively in several respects: “Labrum only” phenotypes were found exclusively in the *black* strain while the abdomen inside-out phenotype always remained below 5%. Moreover, increasing dsRNA concentrations led to a rather mild increase of phenotypic severity but even at highest concentrations the “empty egg” phenotype was not increased beyond background. In the *SB* strain, in contrast, “labrum only” phenotypes were not found at all, while the abdomen inside out class was always high. Increasing amounts of dsRNA led to a significant increase of phenotypic severity leading to 100%“empty egg” phenotypes at high concentrations. At the same time, the phenotypes also show similarities: The pleiotropic defects seen in *SB* and to a minor extent also in *black* represent the expected pleiotropic phenotype of a nuclear import protein.

#### RNAi sensitivity is similar in the *black* and *SB* strains

The different RNAi phenotypes could be due to a different strength of the RNAi response in these strains or alternatively could be due to the different genetic background, which interacted differently with *Tc*-*importin α1* but not other genes. To test this, we first quantified the transcript level in the RNAi animals by qPCR. The expression was reduced by >93% in both strains in both 0-2h, 10-12h egg collections as well as in ovaries (Additional file 1 K). As a complementary means to compare RNAi efficiency in the strains, we quantitatively compared the phenotypic range induced in the *SB* and *black* strains after RNAi using the same dsRNA preparations targeting four different genes.

**Figure 5:**
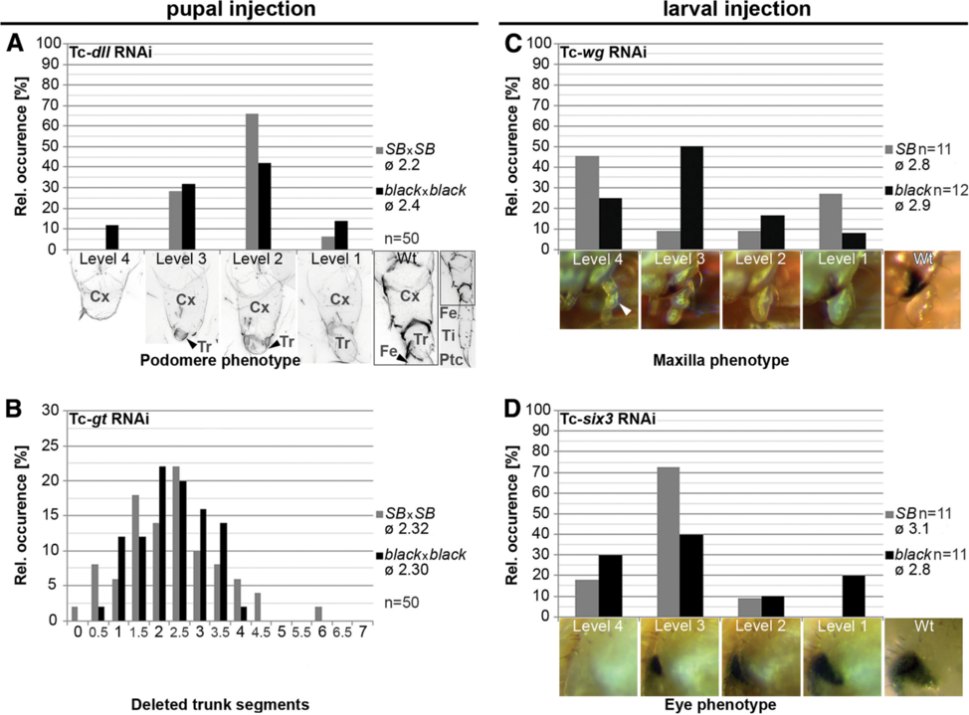
**Similar strength of RNAi in *black *and *SB *strains.** Parental RNAi (pupal injection and analysis of the offspring) is shown for *Tc*-*dll* (**A**) and *Tc*-*giant* (*Tc*-*gt*) (**B**). Larval RNAi was done for *Tc*-*wg* (**C**) and *Tc*-*six3* (**D**). In all panels, the gray bars represent results for *SB* strain while black bars represent the *black* strain. (**A**,**C**,**D**) The phenotypes were grouped into four classes of phenotypic strength represented in the pictures below the panel (Level 1: mildest phenotype; Level 4: strongest phenotype). To calculate the mean phenotypic strength, the phenotype classes were given arbitrary values of 1 to 4 points point for level 1 to level 4. (**B**) In case of *Tc*-*gt*, the number of missing abdominal segments is used for grouping the phenotypes into classes. In all cases, we find a similar distribution of phenotypic strength. Hence, none of the strains appears to be generally more amenable to RNAi than the other in pupal or larval RNAi. *Cx* coxa, *Tr* trochanter, *Fe* femur, *Ti* tibia, *Ptc* pretarsal claw.

First, we checked *Tcdistalless* (*Tcdll*) and *Tcgiant* (*Tcgt*), which elicit well quantifiable defects in the offspring of injected pupae [32,36]. dsRNA targeting *Tcdll* was injected into both strains, 50 cuticles of the first egg collection (d 9 after injection) were analyzed regarding the number and state of the remaining leg segments. The cuticles were grouped into four different classes of phenotypic strength. In the strongest class only a coxa remained present (Level 4; Figure 5A, leftmost panel) while in the weakest class an almost complete trochanter was present (Level 1; Figure 5A, rightmost panel). The strongest phenotype was found only in *black Tcdll* RNAi (Figure 5A, black bars), but also the mildest phenotype was observed more often in the *black* strain. We arbitrarily rated the four categories (one point for level 1 and four points for level 4) and calculated the average phenotype strength, which turned out to be similar (ø *black*: 2.4, ø *SB*: 2.2).

The analogous experiment was performed with *Tc*-*gt* dsRNA (1 μg/μl, n=50). The number of deleted trunk segments was used as measure for the phenotypic strength. This number varied from zero to six deleted trunk segments (Figure 5B). Overall, the distribution in both strains was similar, while both the strongest and the mildest phenotypes were only observed in *SB* cuticles (Figure 5B, gray bars). Also the average of the number of deleted segments was very similar (ø *black*: 2.30, ø *SB*: 2.32).

Further, we performed larval RNAi for *Tc*-*wingless* (*Tc*-*wg*) and *Tc*-*six3* in order to compare the RNAi response after larval RNAi (lRNAi). Knock-down of *Tc*-*wg* via injection of dsRNA (1 μg/μl) into late larval stages (L6) of *SB* and *black* lead to pupae with reduced genital lobes, an increased distance between the pupal wings and a reduced maxillary diameter (Figure 5C). The latter was the best quantifiable indicator because the diameter is very constant in wt pupae (Additional file 1 G). The phenotypic series was divided into four categories (Figure 5C, panel 1–4), was rated and the average was calculated. Both strains show a comparable mean value (ø *black*: 2.9, ø *SB*: 2.8).

*Tc*-*six3* dsRNAi injection (0.5 μg/μl) in L6 larvae led to pupae with reduced eye size (Figure 5D), which was quantified (see experimental procedures). Again, the phenotypes were grouped into four categories and the mean values were calculated (Figure 5D). *Tc*-*six3* lRNAi resulted in slightly stronger pupal phenotypes in the *SB* strain (ø *black*: 2.8, ø *SB*: 3.1).

Taking into account the experimental variability inherent to RNAi experiments, these data suggest that our strains do not have a generally different RNAi response. Moreover, the nucleic acid sequence of the dsRNA fragments is almost identical in both strains (99.4%), making different RNAi efficiencies due to mismatches unlikely. Hence, the strain specific phenotypic difference we observed was likely due to different modulation of the *Tc*-*importin α1* phenotype in the respective genetic backgrounds. This is similar to findings in other model organisms, where the phenotype of mutant alleles of some (but not all) genes is different depending on the genetic background of the strain.

#### Tc-importin α1 peptide sequence is slightly diverged

Next, we asked whether differences of the amino acid composition of Tc-importin α1 protein could be the reason for the differences. We isolated and sequenced the coding sequences of both strains. The Tc-importin α1 amino acid sequences (526 amino acids) of the *black* and *SB* strains were aligned with the sequenced strain (*GA*-*2*) and with Importin α orthologs of other species.

**Figure 6:**
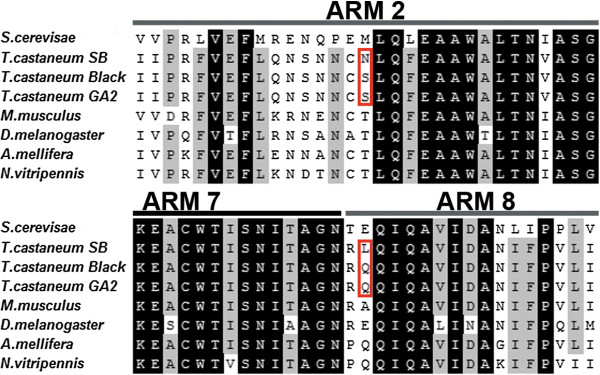
**Amino acid exchanges in the cargo binding domain of Importin α1 between the strains.** Shown is a section of an alignment of Importin α proteins of different species and strains of *Tribolium* including the 2^nd^, 7^th ^and 8^th ^ARM domains. GA2 is the sequenced strain. Black boxes represent identical amino acids, gray boxes denote sites with a conservation of about 90% and horizontal lines indicate the conserved ARM domains involved in cargo binding identified in *S*. *cerevisiae* SRP1 [48]. Red frames indicate two sites in which the amino acid sequence of the *Tribolium* strains differ. Both sites are within the region known to be responsible for cargo binding. Note, that other metazoans show N or S at the respective position within the ARM2 domain (see text for details).

The *black* and the *GA*-*2* strains have identical sequences whereas the *SB* strain has different amino acids at two sites (Figure 6, red frame) within the cargo binding ARM domains. The first site (position 147) is located within the second ARM domain, where the *black* and the *GA*-*2* strains show a serine (S) whereas the *SB* strain shows an asparagine (N). In metazoa, this position is either occupied by a threonine (T) (see examples given in Figure 6), serine (e.g. *Saccoglossus kowalevskii*) or asparagine (e.g. *C*. *elegans*) (not shown). All three amino acids are similar regarding their polar but uncharged side chains.

The second different amino acid is located within the eighth ARM domain (position 376). Here, the *SB* strain encodes a leucine (L, hydrophobic, not polar), whereas the two other *Tribolium* strains show a glutamine (Q, polar uncharged), which is also found in the two hymenopterans *Nasonia vitripennis* (*N*. *vitripennis*) and *Apis mellifera* (*A*. *mellifera*). *M*. *musculus* carries an alanine (A) at this position, which is hydrophobic and not polar, whereas *D*. *melanogaster* has a glutamic acid, which is acidic and negatively charged. None of the two sites is predicted to be the target of phosphorylation, N-glycosalation nor N-myristoylation by ExPASy and Prosite analysis. Taken together, the observed amino acid substitutions may lead to altered binding affinities, which might influence the phenotype. However, this is difficult to test because we do not know which of the many nuclear proteins likely to be imported by Importin αs actually elicit the observed phenotypes. An alternative explanation would be that the mutations lead to differential splicing of the gene in the different strains.

#### The maternal genotype mainly determines the *Tc*-*importin α1* phenotype

Our data hinted at the genetic background as cause for the different phenotypes. However, it remained open whether this would be based on zygotic gene expression (i.e. expression of the embryonic genome) or whether different maternal inputs would be involved (e.g. differential loading of the egg with respective protein or mRNA). In order to distinguish between these possibilities, *Tc*-*importin α1* dsRNA (1 μg/μl) was injected in *SB* and *black* females and these were afterwards mated with males of the other strain, respectively. Therefore, zygotic expression of genes was based on heterozygous condition for *black*/*SB* in all offspring while the maternal contribution was either of the *black* or the *SB* type.

**Figure 7:**
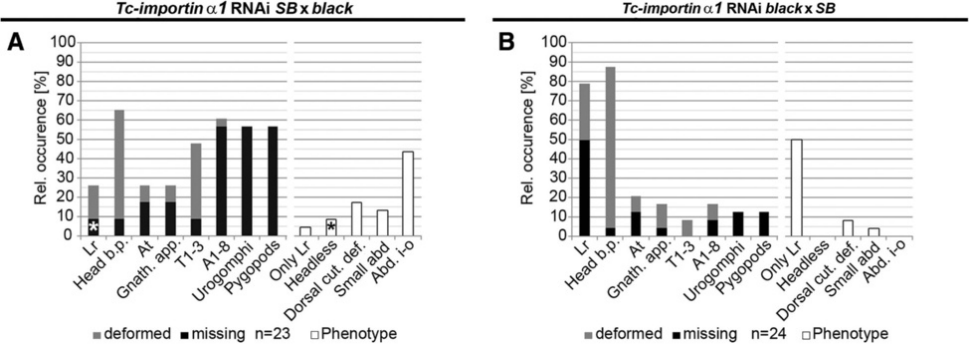
**Maternal genotype determines the cuticle phenotypes.***Tc*-*importin α1* dsRNA was injected into females of one strain, which were then mated to males of the other strain. Hence, the injected strain determines the genotype of the maternal contribution, while the zygotic genetic background is identical in both experiments (heterozygous for *SB* and *black*). (**A**) Injection of dsRNA (1 μg/μl) into female *SB* pupae mated to *black* males resulted in defects similar to those found in the *SB* strain (compare to Figure 2A,B). (**B**) Injection of *Tc*-*importin α1* dsRNA (1 μg/μl) into female *black* pupae crossed with *SB* males resulted in cuticles similar to the *black* strain (compare with Figure 2F,G).

Offspring of injected *SB* females mated with *black* males showed a similar phenotype as described for the *SB* strain (compare Figure 7A with Figure 4B). In 23 analyzed cuticles the “abdomen inside-out” phenotype was most prominent (43.5%). Besides, the “small abdomen” (13%) and the “headless” (8.7%) phenotype could also be observed in some cuticles. The “labrum only” phenotype was very rare (4.3%). When *Tc*-*importin α1* was knocked-down in *black* female pupae which were crossed to *SB* males, the phenotypes were similar to the phenotype of the *black* strain (compare Figure 7B with Figure 4E). The labrum defects were most frequent (79.2%), and in 50% the only affected structure. Also, the *SB* typical “abdomen inside-out” phenotype was not observed.

These results show that it is primarily the genotype of the mother, which determines the quality of the *Tc*-*importin α1* RNAi phenotype while the minor increase of “labrum only” phenotypes to 4,3% indicate some influence of the zygotic genome, too.

The importance of maternal contribution of Importin α proteins is plausible, because it is known from *Drosophila* that the transition from maternal to zygotic control occurs only at cell cycle 13–14 [55,56]. Already before this transition the nuclear import machinery is essential to allow gene activity. This is ensured by a strong maternal contribution [57-59].

#### Relative maternal contribution of *Tc*-*importin α1* is reduced in the *black* strain

Based on the fact that Importin α proteins act redundantly in the import of most proteins [43,46] we asked whether the maternal supply of the oocyte with importin mRNAs would be different in the *SB* and *black* strains. Specifically, we wanted to test the model that the contribution of maternal *Tcimportin α1* was relatively small in the *black* strain and that this was buffered by increased *Tcimportin α2*/*3* contributions. In that case, knock-down of *Tcimportin α1* would lead to less prominent phenotypes in the *black* strain because most defects would be buffered by the other Importins α’s.

**Figure 8:**
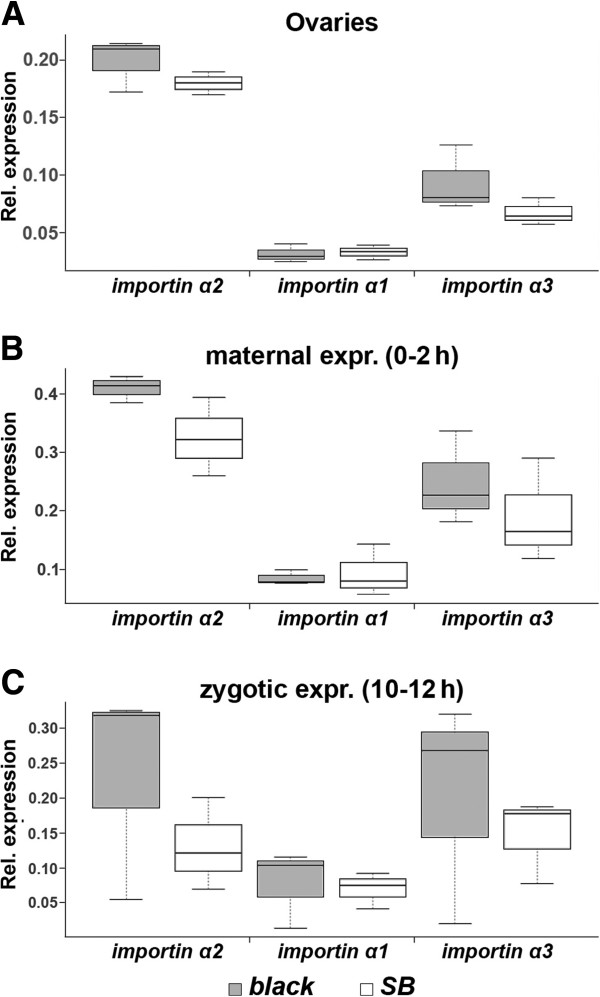
**Expression profile of the three *Tc***-***importin α *genes in the *SB *and the *black *strain.** The relative expression of *Tc*-*importin α* mRNAs was tested in ovaries (where maternal loading of the oocyte takes place; **A**), in freshly laid eggs (representing the maternal contribution; **B**) and at 10-12h of development (zygotic expression; **C**). The boxplot diagrams show that the relative expression of *Tc*-*importin α2* and *Tc*-*importin α3* in the genetic background of *SB* (empty box) tended to be lower in all three tissues/stages compared to their expression in the *black* strain (gray box). However, these results were not statistically significant. See text for further details. Note that for better comparability, the values were normalized such that the *Tc*-*importin α1* expression had the same level in both strains.

By quantitative RT-PCR, we determined the amount of mRNA present in ovaries where maternal load of oocytes takes place, freshly laid eggs which reflect the final maternal load and embryos at 10–12 hours of development, where zygotic transcription has started. The qPCR data were normalized to the expression of the ribosomal protein *TcrpS3* within the samples [60] (Figure 8.). For better comparability between the strains, we subsequently standardized the datasets such that the *Tcimportin α1* levels are identical in both strains (see Materials and Methods). This allowed detecting relative increase or decrease of the other *importin αs*. Both *Tcimportin α2* and *3* levels were higher in all samples in the *black* strain compared to the *SB* strain but none of these differences was significant.

#### Maternal increase of *Tc*-*importin α2* in *Tc*-*importin α1* RNAi embryos in the *black* strain

An alternative possibility for different maternal buffering of the *Tc*-*importin α* paralogs was that the knock-down of *Tc*-*importin α1* would be compensated by different patterns of upregulation of the other *Tc*-*importin α* paralogs in these strains. Indeed, in 0-2h old embryos of *Tc*-*importin α1* RNAi animals, the load of *Tc*-*importin α2* was significantly increased in the *black* but not the *SB* strain (see Additional file 1 K). At this embryonic stage, maternal messages predominate, hence, it appears that maternal upregulation was involved in rescuing parts of the phenotype in the *black* strain. Interestingly, the situation was different when measuring zygotic transcript levels in 10-12h embryos. Here, the *black* transcript levels are not altered much, while in *SB*, *Tc*-*importin α2* and *3* are upregulated. Apparently, the delayed compensation in *SB* is too late to rescue the embryonic phenotype.

#### Potential mechanisms leading to the phenotypic differences

This work was primarily aimed at showing that the quality of RNAi phenotypes may depend on the genetic background of the strain used. However, we also gained some insights into the potential mechanism how the difference might arise in the specific case of *Tcimportin α1*. Two things need to be explained: First, the absence of the expected pleiotropic defect, and second the occurrence of the qualitatively different “labrum only” phenotype in the *black* strain. To explain the apparent decrease of pleiotropic defects, we suggest that females of the *black* strain load their oocytes with more *Tcimportin α2* and *3* relative to *Tcimportin α1* and moreover, compensate for loss of *Tcimportin α1* by upregulating *Tcimportin α2*. Therefore, the importin paralogs are able to rescue the knock-down effect of *Tcimportin α1* much better in the *black* than in the *SB* strain. This would lead to a comparably mild pleiotropic phenotype. Indeed, functional redundancies [61] and the resulting masking of phenotypes [49] by the different Importin α’s was described previously in *Drosophila*.

The *black* specific “labrum only” phenotype might depend on the strain specific amino acid changes found within the cargo binding domains. This difference may have allowed one or several target proteins required for labrum development to evolve an Importin α1 specific import signal. Loss of Tc-Importin α1 would be compensated by the paralogs for most proteins but not for the labrum specific protein, leading to the observed labrum specific phenotype. This model is in line with data showing that Importin paralogs besides their redundant roles in nuclear import of many proteins do also have paralog-specific cargoes [54,62]. A prerequisite for testing this model is the identification of all genes that lead to “no labrum” phenotypes. Then, these could be tested for differential binding with the different Importin αs.

Alternatively, a higher relative expression of *Tc*-*importin α1* in the labrum anlagen of the *black* strain could contribute to a “labrum only” phenotype. However, we were not able to detect differences in labral expression of *Tc*-*importin α1* in the *black* and *SB* strains by in situ hybridization. We might have missed mild modulations of expression–whether such minor differences would be able to lead to such a clear phenotype remains questionable. However, with the current data, we cannot exclude that the mechanism is much more complex and may involve many additional factors and interactions.

## Conclusions

### Documentation of strains is essential for future RNAi studies

It has been known from mice and *Drosophila* that different genetic backgrounds of laboratory inbred strains can affect the phenotypes in transgenic experiments (e.g. [6-8,63-65]. This may be due to changes within coding or non-coding regions [66]. Recently, Dworkin et al. argued that strain specific modulation of phenotypes may have to be considered more systematically than in the past [67]. Here, we show that this is also true for RNAi studies, which to our knowledge has not been considered in the past. Our findings have implications for the increasing number of RNAi experiments in an increasing number of animal taxa. First, discrepancies of results between labs might be due to the use of different strains. Second, the strain used in an RNAi experiment needs to be documented and kept over time to allow the reproduction of the phenotypes by others. Third, confirming the results of an RNAi experiment in another strain provides a good means to test for the general relevance of a phenotype.

### RNAi as tool for studying genetic differences on the population level

Our finding also opens new possibilities. The ease of application of RNAi allows systematically identifying differences in gene regulatory networks between populations of one species including species that cannot be kept in the lab. Such changes provide the genetic variability, which is required for the evolution of novel traits. RNAi will allow a systematic investigation of the degree of variability within species.

## Methods

### Cloning

*Tc*-*importin α1* open reading frame sequence (1581 bp; accession: [XM_963412]) was obtained from the iBeetle genome browser (http://bioinf.uni-greifswald.de/gb2/gbrowse/tcas/). The following primers were used to amplify the open reading frame froman embryonic cDNA pool (0-48 h) via standard PCR: 5’-ATGTCGGGCTCCGCTCACAA-3’ and 5’-TTAAAAATGGAATCCTCCCATCGGCACCG -3’. The *Tc*-*importin α1* open reading frame was cloned into the pJET1.2 vector.

### RNAi

The sequences of the fragments used for RNAi are given in the Additional file1 B. The templates for the non-overlapping fragments were generated by PCR from a plasmid template using following primers: 5’-TAATACGACTCACTATAGGAGTCTGGAGGAGGGTTCTTGC-3’ and T7 Primer (5’-TAATACGACTCACTATAGG-3’) for the 5’ fragment (*Tcimportin α1*a, 709 bp; see Additional file 1 A and B, gray bar) and 5’-TAATACGACTCACTATAGGTTGCGAAAGTCTCCCCAGCT-3’ and pJET1.2R sequencing primer with a T7-attachment (5’-TAATACGACTCACTATAGGAAGAACATCGATTTTCCATGGCAG-3’) for the 3’ fragment (*Tcimportin α1*b, 872 bp; see Additional file 1 A and B, black bar). Concentrations for parental RNAi were 0.3 μg/μl, 1 μg/μl and 3 μg/μl (*Tcimportin α1*a and *Tcimportin α1*b), 1 μg/μl (*Tcgt* and *Tcdll*) and for larval RNAi 1 μg/μl (*Tcwg*) and 0.5 μg/μl (*Tcsix3*). Pupal injections were performed as described [32]. For larval RNAi (lRNAi) the larvae were anaesthetized by cooling them on ice. The dsRNA was injected using the FemtoJet express (eppendorf, Hamburg). Late larval stages (L6) were injected into the ventro-lateral side between the fifth and sixth abdominal segment. On average 0.4-0.5 μl dsRNA were injected into one larva. Injected larvae were raised as described [34].

### Microscopy and Image analysis

Cuticles were documented using a Zeiss LSM 510 as described [68,69]. *Tcdll* RNAi legs were recorded in 15 focal planes using a Zeiss Axioplan microscope and Image-Pro Plus software (MediaCybernetics^®^, version 6.2). Deconvolution was performed with the “No Neighbour” method followed by a maximum projection using ImageJ (version 1.44 o). Pupae were analyzed and documented using a Leica M205 FA fluorescence stereomicroscope.

lRNAi pupae were analyzed using ImageJ (version 1.44 o). The diameter of the second segment of the maxillary palpus (Figure 5C, white arrowhead) was measured using the straight line tool. Division in phenotypic levels: Level 1= wild-type (wt) range of diameter, level 2= minimum wt diameter minus wt range, level 3= minimum level 2 diameter minus wt range, level 4= minimum level 3 diameter minus wt range. The eye field area of both eyes in *Tc*-*six3* lRNAi in late pupal stages (fully sclerotized mandibles) was measured by freehand selection tool and the mean was calculated. The mean value provided the basis for the phenotype comparison. The phenotype level was chosen arbitrarily.

### Phylogenetic analysis

Importins of the different species were obtained using amino acid sequence of Tc-Importin α1 as query for a BLASTp search [70] at NCBI (http://blast.ncbi.nlm.nih.gov/Blast.cgi, Figure S2). Phylogenetic analysis was conducted using *MEGA* version 5 [71]. The multiple alignment was done using the ClustalW application with the preset parameters. A phylogenetic tree was calculated using the Neighbor-joining method under the Poisson amino acid substitution model. Bootstrap analysis was conducted using 1000 replicates to test the robustness of the phylogenetic tree. Calculation of a phylogenetic tree using the Maximum-Likelihood method under the Jones-Taylor-Thornton model amino acid substitution model results in essentially the same phylogenetic tree.

### Correspondence analysis

For correspondence analysis [72], the labrum, head bristle pattern, antennae, gnathal appendages, thoracal segments, abdominal segments, pygopods and urogomphi of each L1 cuticle were classified into three different categories: not affected=0, deformed=0.5 and absent=1. The dataset was imported into R (v. 2.14.2, [73]) and correspondence analysis and plotting was performed by using the R ‘ca’ package [74].

### Quantitative RT-PCR

Total RNA was isolated from dissected ovaries of adult beetles using the Tissue & Insect RNA MicroPrep™ Kit (Zymo Research Corporation, Irvine) and from eggs (0–2 h and 10–12 h) using TRIzol^® ^reagent (Ambion^®^/Live technologies, New York). 1 μg/μl total RNA was converted to cDNA by using the MAXIMA^® ^First Strand cDNA Synthesis Kit for RT-qPCR (Thermo Scientific, Waltham). Quantitative PCR was performed using HOT FIREPol^® ^EvaGreen^® ^qPCR Mix Plus (ROX) (Solis BioDyne, Tartu) and the CFX96™ Real-Time PCR System (Bio-Rad Laboratories, Hercules). For the qRT-PCR the following primer pairs were used: *Tc*-*importin α1*: 5’-CCGTATGCTGTGCTAATCGAG-3’ and 5’-CGTCCCGAAGAAGTGTTCAAT-3’, *Tc*-*importin α2*: 5’-AAAGTCTACGAACGGGCTTTG-3’ and 5’-GAACTGAATCTCCCCATTTGC-3’, *Tc*-*importin α3*: 5’-TGAGGAGTGCAATGGCTTAGA-3’ and 5’-TCATCCGCATCACCACTAAAG-3’ and *Tc*-*rpS3*: 5’-ACCTCGATACACCATAGCAAGC-3’ and 5’-ACCGTCGTATTCGTGAATTGAC-3’. All primers were designed to span an intronic sequence and were validated by gel analysis. To calculate primer efficiency (E=10^(−1/m)), a dilution series was performed. Data was normalized by the formula: rel. expression=R_target_^Cq_target_/R_ref._^Cq_ref_. For better comparison of the results in the two strains, the expression levels were normalized such that the genes with the lowest relative expression were set to the same values in the *SB* and *black* strains. Specifically, the difference between the means of *Tc*-*importin α1* expression levels in the *SB* and the *black* strains were calculated. Subsequently, all *SB* expression levels were reduced by this mean difference (Figure 8). This normalization was not done for Additional file 1 K. For statistical analysis, three tests were performed: Welch’s t-test, Student’s t-Test and the Mann–Whitney-U-test. None of these tests revealed significant differences between the respective expression profiles in *SB* and *black*.

## Competing interests

The authors declare that they have no competing interests.

## Authors’ contributions

JS identified the phenotype in the iBeetle screen, PK did the experiments, analyzed the data and wrote the manuscript, CSE contributed to the design of the study and the analysis of the data, GB conceived of the study, analyzed the data and finalized the manuscript. All authors read and approved the final manuscript.

## Supplementary Material

Additional file 1 **Genomic structure of *Tc***-***importin α1*, dsRNA fragments, alignments, tables with cuticle analyses, *Tc*-*importin α1 *RNAi phenotype in the *GA*-*2 *and *pig19 *strains, qPCR of importin paralogs in *Tc*-*importin α1 *RNAi.** Click here for file
