# Supplementary material for: RNAi phenotypes are influenced by the genetic background of the injected strain
Source: BMC Genomics. 2013 Jan 16;14:5. doi: 10.1186/1471-2164-14-5 (PMC3574008; doi:10.1186/1471-2164-14-5)
Supplement: Additional file 1 — Genomic structure of Tc-importin α1, dsRNA fragments, alignments, tables with cuticle analyses, Tc-importin α1 RNAi phenotype in the GA-2 and pig19 strains, qPCR of importin paralogs in Tc-importin α1 RNAi. [file 1471-2164-14-5-S1.pdf]

**Additional file 1**

Kitzmann et al.

**A) Genomic structure of *Tc-importin α1 (Tc000963*) and location of the dsRNA fragments**

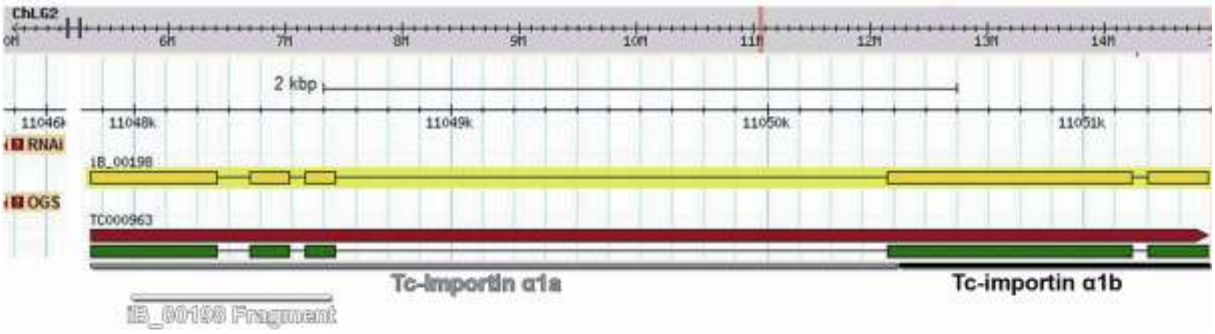


*TC000963* (NCBI LOC656915) is located on linkage group 2. The iB_00198 dsRNA targets the first three exons (white bar). The *Tc-importin α1*a dsRNA fragment targets in addition also a part of the fourth exon sequence (grey bar) and the *Tc-importin α1*b dsRNA fragment targets the fourth and fifth exon (black bar).

***B) Tc000963* open reading frame and dsRNA fragments used in RNAi experiments**

The iB_00198 fragment is underlined, the non-overlapping dsRNA fragments *Tc-importin α1*a and *-importin α1*b are highlighted in italic and regular fonts, respectively.

*ATGTCGGGCTCCGCTCACAAACACCGCTACAAAAACGCGGCGCTGGACAGCGTCGAGTTGCGACGGC*
*GGCGAGAGGAAGAAGGCCTCCAGTTAAGGAAACAGAAACGGGAACAGCAGCTGAACAAGCGGCGG*
*AATGTTAACGTCAACCAATTGGCCGAGGACAATGACCATCTGCAAGTGGACAGCGAGATCCTCTCGC*
*CTGTCTCGCCCATGATCACCCCCGAAATGGTCCAGGCGTTGTACAGCCCCGACGTGGAGCAGCAAATC*
*AGCGCCACGCAAAAATTCAGACAACTGCTGAGCTACGAACCCAACCCACCCATTGATGAGGTGGTCC*
*AGACCGGGATCATCCCACGATTTGTAGAGTTTTTGCAGAATTCAAACAATTGTAGCCTTCAGTTTGAA*
*GCAGCATGGGCTTTGACCAACGTTGCCTCTGGTACCTCTCAGCAAACCAGGATGGTCATTGAAGCTG*
*GTGCTGTTCCTATTTTTATACGTTTGTTGAGCAGTCAATATGAGGATGTTCAGGAGCAAGCTGTTTGG*
*GCATTAGGGAATATAGCAGGGGATTCACCCGAATGTAGAGATCACGTCCTGGATTCGGGTATTTTGG*
*TTCCACTCTTACAACTCCTTAGTAAATCGACGCGTTTATCGATGACTCGCAATGCCGTGTGGGCGCTTT*
*CGAACCTCTGCAGGGGCAAGAACCCTCCTCCAGACT*TTGCGAAAGTCTCCCCAGCTCTGCCCGTACTC
GCTCGTCTCTTGTTTCACTCCGACCCTGACGTGCTCTCTGACACTTGCTGGGCCTTGAGCTACCTATCC
GACGGTCCTAACGAAAAAATCCAGGCGGTCATAGACGCCGGCGTTTGTCGGAAACTGGTCGAATTG
CTAATGCACCAACAGCCCAACGTAGTCTCGGCAGCTCTGCGCGCTGTCGGCAACATAGTGACTGGTG
ACGACGTCCAAACTCAGGTCATACTAAACTGCTCCGCTCTACATTGCTTGCACCACTTGTTGTCATCCT
CGAAGGAGTCCGTGAGGAAGGAGGCGTGTTGGACGATCAGCAACATCACGGCTGGAAACCGGCAG
CAAATCCAGGCGGTCATAGACGCGAACATTTTCCCGGTTTTGATAGAGATTTTGAGCAAGGCGGAGT

TTAAGACGCGGAAGGAGGCGGCCTGGGCTATCACGAATGCCACCAGCGGGGGCACACCCGACCAG
ATCAGGTATCTAGTGAACCAGGCTTGCATTGGGCCGCTTTGTGACCTTTTGACCGTCATGGACGCGA
AAATCGTGCAAGTGGCCCTCAACGGCCTGGAGAATATTCTGAGATTAGGGGAGCAAGACGCCAAGA
ATCACTCGGGGACCAACCCGTATGCTGTGCTAATCGAGCAGTGCTATGGGTTGGATAAAATCGAATT
TTTGCAATCGCATGTCAATATGGAGATTTATCAGAAGGCCTTCGACATTATTGAACACTTCTTCGGGA
CGGAGGAGGAGGACACGACTGTGGCGCCCTCGGTGAATCCCGACCAACAGCAGTACCATTTCAGCT
CGGATCAGTCGGTGCCGATGGGAGGATTCCATTTTTAA

## C) Fasta sequences of the aligned amino acid sequences of Importin α1 orthologs

>*D.m.*/Importin α1

MSA-
HKKNAALDSTEMRRRREEVGIQLRKNKREQQLFKRRNVVLEPNEQQAADMHMADSNEEMIQMLFSGRENEQLEATQKFRKLLSRDPNPPIE
EVIQKGIVPQFVTFLRNSANATLQFEAAWTLTNIASGTSQQTKVVIEAGAVPIFIDLLSSPHDDVQEQAVWALGNIAGDSPMCRDHLLGSGILE
PLLHVLSNSDRITMIRNAVWTLSNLCRGKSPPADFAKISHGLPILARLLKYTDADVLSDTCWAIGYLSDGPNDKIQAVIDAGVCRRLVELLLHPQ
QNVSTAALRAVGNIVTGDDQQTQVILGYNALTCISHLLHSTAETIKKESCWTISNIAAGNREQIQALINANIFPQLMVIMQTAEFKTRKEAAWAI
TNATSSGTHEQIHYLVQVGCVPPMCDFLTVVDSDIVQVALNALENILKAGEKFQTPYAITIEECGGLDKIEYLQAHENRDIYHKSFYIIEQYFGEEE
DSRVAPVAG--TQQFEFDPDN-MPSTGFNF

>*D.m.*/Importin α2

MSKDSNKANSINTQDSRMRRHEVTIELRKSKKEDQMFKRRNIN-
DEPLKELNGQSPVQLVDEIVAAMNSEDQERQFLGMQSARKMLSRERNPPIDLMIGHGIVPICIRFLQNTNNSMLQFEAAWALTNIASGTSDQ
TRCVIEHNAVPHFVALLQSKSMNLAEQAVWALGNIAGDGAAARDIVIHHNVIDGILPLINNETPLSFLRNIVWLMSNLCRNKNPSPPFDQVKRL
LPVLSQLLLSQDIQVLADACWALSYVTDDDNTKIQAVVDSDAVPRLVKLLQMDEPSIIVPALRSVGNIVTGTDQQTDVVIASGGLPRLGLLLQH
NKSNIVKEAAWTVSNITAGNQKQIQAVIQAGIFQQLRTVLEKGDFKAQKEAAWAVTNTTTSGTPEQIVDLIEKYILKPFIDLLDTKDPRTIKVVQT
GLSNLFALAEKLGGNLCLMVEEMGGLDKLETLQQHENEEVYKKAYAIIDTYFSGDDEAEQELAPQEVNGALEFNATQPAPEGGYTF

>*D.m.*/Importin α3

--
MSMEKNKGKDQDEMRRRRNEVTVELRKNKREETILKRRNVPNLDTDEEEQLSSSIDLKKLAKAAADATKPEQQLAAVQAARKLLSSDKNPPIN
DLIQSDILPILVECLKQHNHTMLQFEAAWALTNIASGTSAQTNEVVAAGAVPLFLQLLNSPAPNVCEQAVWALGNIIGDGPLLRDFVIKHGVVQ
PLLSFIKPDIPITFLRNVTWVIVNLCRNKDPAPPTATIHEILPALNVLIHHTDTNILVDTVWAISYLTDGGNDQIQMVIESGVVPKLIPLLGNSEVKV
QTAALRAVGNIVTGSDEQTQVVLNYDALSYFPGLLSHPKEKIRKEAVWFLSNITAGNQSQVQAVINVGLLPKIIENLSKGEFQTQKEAAWAISNL
TISGNREQVFTLIKEGVIPPFCDLLSCQDTQVINVVLDGLNNMLKVADSHVEAVANCIEECEGLAKIERLQSHENVEIYKLAYEIIDQYFTEGEQTN
MAPTSD-GAQ-YNFDPHADLTMNSFNF

>*M.m.*/Karyopherin α1

MSTGKEKNKSLNPDEMRRRRREEEGLQLRKQKREEQLFKRRNVATAEEEVMSDGFEAINNTSDMTDMIFSNSPEQQLSATQKFRKLLSKEPNP
PIDEVINTGVVARFVEFLKRKENCTLQFESAWVLTNIASGNSLQTRNVIQAGAVPIFIELLSSEFEDVQEQAVWALGNIAGDSTMCRDYVLNCNI
LPPLLQLFSKQNRLTMTRNAVWALSNLCRGKSPPPEFAKVSPCLNVLSWLLFVSDTDVLADACWALSYLSDGPNDKIQAVIDAGVCRRLVELL
MHNDYKVVSPALRAVGNIVTGDDIQTQVILNCSALQSLLHLLSSPKESIKKEACWTISNITAGNRAQIQTVIDANMFPALISILQTAEFRTRKEAA
WAITNATSGGSAEQIKYLVELGCIKPLCDLLTVMDAKIVQVALNGLENILRLGEQEAKPYCALIEEAYGLDKIEFLQSHENQEIYQKAFDLIEHYFG
EDEDSSIAPQVDLSQQQYIFQQCE-APMEGFQL

>*M.m.*/Importin α2

MATKAPKYRGKEMSLPRQQRIASSLQLRKTRKDEQVLKRRNIDLFSDMVSQALVKEVNFLDDIIQAVNSSDPILHFRATRAAREMISQENTPPL
NLIIEAGLIPKLVDFLKATPHPKLQFEAAWVLTNIASGTSEQTRAVVKEGAIQPLIELLCSPHLTVSEQAVWALGNIAGDCAEFRDCVISNNAIPHL
INLISKGIPITFLRNISWTLSNLCRNKDPYPSESAVRQMLPPLCQLLLHRDNEILADTCWALSYLTKGGKEYIHHVVTTGILPRLVELMTSSELSISIP

CLHTIGNIVAGTDEQTQMAIDAGMLKVLGQVLKHPKTSIQVLAAWTMSNVAAGPRHQVEQLLCN-
LLPILVDLLRNAELKVQKEVVCTVINIATGASQDQLTLLAHSGILEPMLSLLSAPDLEVVIIVLDIISYLLQHIDNLQERLYFQIEKFGGFEKIECLQHH
HNISISNSALDIIEKYFC-DGDGDSLPGPGLRV------------------

>*M.m.*/Karyopherin α3

AENGLEKNKGRDVETMRRHRNEVTVELRKNKRDEHLLKKRNVPQEEDSDVDADFKAQNVLEAILQNATSDNPVVQLSAVQAARKLLSSDRN
PPIDDLIKSGILPILVKCLERDDNPSLQFEAAWALTNIASGTSAQTQAVVQSNAVPLFLRLLHSPHQNVCEQAVWALGNIIGDGPQCRDYVISLG
VVKPLLSFINPSIPITFLRNVTWVIVNLCRNKDPPPPMETVQEILPALCVLIYHTDINILVDTVWALSYLTDGGNEQIQMVIDSGVVPFLVPLLSHQ
EVKVQTAALRAVGNIVTGTDEQTQVVLNCDVLSHFPNLLSHPKEKINKEAVWFLSNITAGNQQQVQAVIDAGLIPMIIHQLAKGDFGTQKEAA
WAISNLTISGRKDQVEYLVQQNVIPPFCNLLSVKDSQVVQVVLDGLKNILIMAGDEASTIAEIIEECGGLEKIEVLQQHENEDIYKLAFEIIDQYFSI
DEDPSLIPEAT-QGGTYNFDPTANLQTKEFNF

>*M.m.*/Karyopherin α4

ADNKLDKNKGRDLETMRRQRNEVVVELRKNKRDEHLLKRRNVPQEDDSDIDGDYRVQNTLEAIVQNASSDNQGIQLSAVQAARKLLSSDRN
PPIDDLIKSGILPILVHCLERDDNPSLQFEAAWALTNIASGTSEQTQAVVQSNAVPLFLRLLHSPHQNVCEQAVWALGNIIGDGPQCRDYVISLG
VVKPLLSFISPSIPITFLRNVTWVMVNLCRHKDPPPPMETIQEILPALCVLIHHTDVNILVDTVWALSYLTDAGNEQIQMVIDSGIVPHLVPLLSH
QEVKVQTAALRAVGNIVTGTDEQTQVVLNCDALSHFPALLTHPKEKINKEAVWFLSNITAGNQQQVQAVIDANLVPMIIHLLDKGDFGTQKE
AAWAISNLTISGRKDQVAYLIQQNVIPPFCNLLTVKDAQVVQVVLDGLSNILKMAEDQAETIANLIEECGGLEKIEQLQNHENEDIYKLAYEIIDQ
FFSIDEDPSLVPESV-QGGTFGFNSSTNVPTEGFQF

>*M.m.*/Karyopherin α2

MSTENAKNKGKDSTEMRRRRIEVNVELRKAKKDEQMLKRRNVSSFPPLQENNNQGTVNWVEDIVKGINSNNLESQLQATQAARKLLSREKQ
PPIDNIIRAGLIPKFVSFLGKTDCSPIQFESAWALTNIASGTSEQTKAVVDGGAIPAFISLLASPHAHISEQAVWALGNIAGDGSAFRDLVIKHGAI
DPLLALLAVPLACGYLRNLTWTLSNLCRNKNPAPPLDAVEQILPTLVRLLHHNDPEVLADSCWAISYLTDGPNERIEMVVKKGVVPQLVKLLGA
TELPIVTPALRAIGNIVTGTDEQTQKVIDAGALAVFPSLLTNPKTNIQKEATWTMSNITAGRQDQIQQVVNHGLVPFLVGVLSKADFKTQKEAA
WAITNYTSGGTVEQIVYLVHCGIIEPLMNLLSAKDTKIIQVILDAISNIFQAAEKLGEKLSIMIEECGGLDKIEALQRHENESVYKASLNLIEKYFSEEE
DQNVVPETTSEGFAFQVQDGAP---GTFNF

>*M.m.*/Karyopherin α6

MASGKDKNNALNPEEMRRRREEEGIQLRKQKREQQLFKRRNVELINEAAMFDLLDSVSSTREMVEMLFSDDSDLQLATTQKFRKLLSKEPSPP
IDEVINTGVVDRFVEFLKRNENCTLQFEAAWALTNIASGTSQQTKIVIEAGAVPIFIELLNSDFEDVQEQAVWALGNIAGDSSLCRDYVLNCSILN
PLLTLLTKSTRLTMTRNAVWALSNLCRGKNPPPEFAKVSPCLPVLSRLLFSSDSDLLADACWALSYLSDGPNEKIQAVIDSGVCRRLVELLMHND
YKVASPALRAVGNIVTGDDIQTQVILNCSALPCLLHLLSSSKESIRKEACWTISNITAGNRAQIQAVIDANIFPVLIEILQKAEFRTRKEAAWAITNA
TSGGTPEQIRYLVSLGCIKPLCDLLTVMDSKIVQVALNGLENILRLGEQESKPYCGLIEEAYGLDKIEFLQSHENQEIYQKAFDLIEHYFGEDDDSSL
APQVDETQQQFIFQQPE-APMEGFQL

>*T.c.*/Importin α1

MSGAHKKNAALDSVELRRRREEEGLQLRKQKREQQLNKRRNVNVNQNDLQDSILSPVSPTPEMVQALYSPDVEQQISATQKFRQLLSYEPNP
PIDEVVQTGIIPRFVEFLQNSNNCSLQFEAAWALTNVASGTSQQTRMVIEAGAVPIFIRLLSSQYEDVQEQAVWALGNIAGDSPECRDHVLDS
GILVPLLQLLSKSTRLSMTRNAVWALSNLCRGKNPPPDFAKVSPALPVLARLLFHSDPDVLSDTCWALSYLSDGPNEKIQAVIDAGVCRKLVELL
MHQQPNVVSAALRAVGNIVTGDDVQTQVILNCSALHCLHHLLSSSKESVRKEACWTISNITAGNRQQIQAVIDANIFPVLIEILSKAEFKTRKEA
AWAITNATSGGTPDQIRYLVNQACIGPLCDLLTVMDAKIVQVALNGLENILRLGEQDAKPYAVLIEQCYGLDKIEFLQSHVNMEIYQKAFDIIEH
FFGEEEDTTVAPSVNPDQQQYHFSSDQSVPMGGFHF

>*T.c.*/Importin α2

--
MDENKNKGKDCEEMRRRRAGQTIELRKAKKEDQLLKRRNISLQEPLQENNSVSLITMAEEILMGMMSPDENLQFKATQACRKMLSRERNPPI
DHMIRLGVVPRCVEFLGKTHNPSLVFEACWALTNIASGTSEQTAAVVQEGALPKLQQLLSSNRIDVVEQAIWAIGNIAGDGPESRDLVLNYGVL
PSLINLIKPNTTLSLLRNTVWVISNLCRNKNPYPDFELVKPALPVLARLLSHDDKDVLADTCWALSYLTDGSNEKIQAVLDTGLIDRLVMLLYSEES
TVLTPALRAVGNIVTGNDHQTDMVINAGALDCMIRLLQHSRLNIVKEAAWTVSNITAGNSEQIQKVLDAGILPYLLHVLQTGDFKSQKEAAWA
VTNFTSGGNSAQLAQLVEMGALKPMCNLLNSKDSKTIIVVLEGLNNILSAAAKLNQKVAIMIEECGGLDGIEALQSHDNEKVYERALHIIENYFA
GEEGVTVTVANGEIQFATPNMADQQSP---FSF

>*T.c.*/Importin α3

GQVGINKNKGKDQDEMRRRRNEVTVELRKNKREETLQKRRNVPVVDEDDIEKSLSTTNLEELVAQAAAIDNPPLQLMAIQTARKLLSSDRNPP
IDALISSGILPVLVMCLERYDEPSLQFEAAWALTNIASGTSAQTNRVVQAGAVPLFLKLLHSPQQNVCEQAVWALGNIIGDGPQLRDYVIELGV
VNPLLSFIKPDVPISFLRNVTWVIVNLCRNKDPPPPIQTIRELLPALNALIHHTDTNILVDTVWALSYLTDGGNEQIQMVIDSGVVPKLIPLLSHKE
VKVQTAALRAVGNIVTGTDEQTQVVLNCDALSHFPALLSHSKEKICKEAVWFLSNITAGNQMQVQAVIDAGLLPNIINNLSKGDFQTQKEAA
WAISNLTIGGNKEQVATLIREGVIPPFCDLLSCKDAQVIQVVLDGINNMLKMAGAEVDQLCTMIEECNGLDKIEMLQNHDNIEIYKIAYDIIEQY
FSADDDANLAPTAA-ETG-FQFDPNTNVPNEGFKF

## D) Cuticle defects after *Tc-importin α1*a RNAi in *black* female pupae mated to *pig19* males

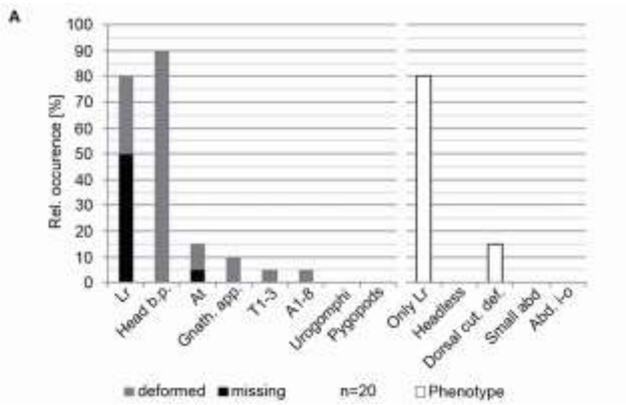


The *Tc-importin α1a* fragment was used for RNAi (1 µg/µl) in the strain combination, which was used in the iBeetle screen. This resulted in 80.0% with exclusive labrum defects which are either marked by a complete loss (Fig 2I, J) or deformation (Fig 2H) of the labrum. The next most frequently affected structure is the antenna, which shows defects in 15% of all cuticles. *Lr* labrum, *Head b.p.* Head bristle pattern, *At* antennae, *Gnath. app.* gnathal appendages, *T1-3* thoracic segment 1-3, *A1-8* abdominal segments 1-8, *Dorsal cl.* dorsal closure, *Abd. i-o* abdominal segments.

## E) Tables summarizing the defects found in the *Tc-importin α1* RNAi experiments

### Cuticle defects after injection of *Tc-importin α1*a dsRNA (1 µg/µl) in *black* female pupae which were crossed to *pig19* males.

| ID | impα1a Blxpig |
|---|---|
| dsRNA[µg/µl] | 1 |
| n | 21 |
| wt | 1 |
| pt | 20 |
| % affected | 95.2 |

| | Lr | Head b.p. | At | Gnath. app. | T1-3 | A1-8 | Urogomphi | Pygopods |
|---|---|---|---|---|---|---|---|---|
| missing | 10 | 0 | 1 | 0 | 0 | 0 | 0 | 0 |
| deformed | 6 | 18 | 2 | 2 | 1 | 1 | 0 | 0 |
| ∑ | 16 | 18 | 3 | 2 | 1 | 1 | 0 | 0 |
| % ∑ | 80.0 | 90.0 | 15.0 | 10.0 | 5.0 | 5.0 | 0 | 0 |
| % missing | 50.0 | 0 | 5.0 | 0 | 0 | 0 | 0 | 0 |
| % deformed | 30.0 | 90.0 | 10.0 | 10.0 | 5.0 | 5.0 | 0 | 0 |

| | Only Lr | Headless | Dorsal closure | Small abd | Abd i-o |
|---|---|---|---|---|---|
| n | 16 | 0 | 3 | 0 | 0 |
| % | 80.0 | 0 | 15.0 | 0 | 0 |

### Cuticle defects after injection of *Tc-importin α1*a dsRNA (1 µg/µl) in *SB* female pupae which were crossed to *SB* males.

| ID | impα1a SBxSB |
|---|---|
| dsRNA[µg/µl] | 1 |
| n | 30 |
| wt | 0 |
| pt | 30 |
| % affected | 100 |

| | Lr | Head b.p. | At | Gnath. app. | T1-3 | A1-8 | Urogomphi | Pygopods |
|---|---|---|---|---|---|---|---|---|
| missing | 4 | 4 | 8 | 12 | 5 | 17 | 15 | 15 |
| deformed | 10 | 19 | 4 | 8 | 18 | 11 | 3 | 5 |
| ∑ | 14 | 23 | 12 | 20 | 23 | 28 | 18 | 20 |
| % ∑ | 46.7 | 76.7 | 40.0 | 66.7 | 76.7 | 93.3 | 60.0 | 66.7 |
| % missing | 13.3 | 13.3 | 26.7 | 40.0 | 16.7 | 56.7 | 50.0 | 50.0 |
| % deformed | 33.3 | 63.3 | 13.3 | 26.7 | 60.0 | 36.7 | 10.0 | 16.7 |

| | Only Lr | Headless | Dorsal closure | Small abd | Abd i-o |
|---|---|---|---|---|---|
| n | 0 | 4 | 7 | 4 | 11 |
| % | 0 | 13.3 | 23.3 | 13.3 | 36.6 |

### Cuticle defects after injection of *Tc-importin α1*b dsRNA (1 µg/µl) in *SB* female pupae which were crossed to *SB* males.

| ID | impα1b SBxSB |
|---|---|
| dsRNA[µg/µl] | 1 |
| n | 13 |
| wt | 0 |
| pt | 13 |
| % affected | 100 |

| | Lr | Head b.p. | At | Gnath. app. | T1-3 | A1-8 | Urogomphi | Pygopods |
|---|---|---|---|---|---|---|---|---|
| missing | 0 | 0 | 1 | 0 | 1 | 9 | 8 | 8 |
| deformed | 3 | 9 | 3 | 4 | 7 | 4 | 0 | 0 |
| ∑ | 3 | 9 | 4 | 4 | 8 | 13 | 8 | 8 |
| % ∑ | 23.1 | 69.2 | 30.8 | 30.8 | 61.5 | 100.0 | 61.5 | 61.5 |
| % missing | 0 | 0 | 7.7 | 0 | 7.7 | 69.2 | 61.5 | 61.5 |
| % deformed | 23.1 | 69.2 | 23.1 | 30.8 | 53.8 | 30.8 | 0 | 0 |

| | Onyl Lr | Headless | Dorsal closure | Small abd | Abd i-o |
|---|---|---|---|---|---|
| n | 0 | 0 | 3 | 2 | 6 |
| % | 0 | 0 | 23.1 | 15.4 | 46.2 |

## Cuticle defects after injection of *Tc-importin α1*a dsRNA (1 µg/µl) in *black* female pupae which were crossed to *pBa19* males.

| ID | impα1a BlxBl |
|---|---|
| dsRNA[µg/µl] | 1 |
| n | 26 |
| wt | 4 |
| pt | 22 |
| % affected | 84.6 |

| | Lr | Head b.p. | At | Gnath. app. | T1-3 | A1-8 | Urogomphi | Pygopods |
|---|---|---|---|---|---|---|---|---|
| missing | 11 | 0 | 2 | 1 | 0 | 1 | 1 | 1 |
| deformed | 8 | 17 | 3 | 4 | 0 | 0 | 0 | 0 |
| ∑ | 19 | 17 | 5 | 5 | 0 | 1 | 1 | 1 |
| % ∑ | 86.4 | 77.3 | 22.7 | 22.7 | 0 | 4.5 | 4.5 | 4.5 |
| % missing | 50.0 | 0 | 9.1 | 4.5 | 0 | 4.5 | 4.5 | 4.5 |
| % deformed | 36.4 | 77.3 | 13.6 | 18.2 | 0 | 0 | 0 | 0 |

| | Only Lr | Headless | Dorsal closure | Small abd | Abd i-o |
|---|---|---|---|---|---|
| n | 14 | 0 | 0 | 0 | 1 |
| % | 63.6 | 0 | 0 | 0 | 4.5 |

**Cuticle defects after injection of *Tc-importin α1*b dsRNA (1 µg/µl) in *black* female pupae which were crossed to *black* males.**

| ID | impα1b BlxBl |
|---|---|
| dsRNA[µg/µl] | 1 |
| n | 9 |
| wt | 2 |
| pt | 7 |
| % affected | 77.8 |

| | Lr | Head b.p. | At | Gnath. app. | T1-3 | A1-8 | Urogomphi | Pygopods |
|---|---|---|---|---|---|---|---|---|
| missing | 3 | 0 | 0 | 0 | 0 | 0 | 0 | 0 |
| deformed | 3 | 5 | 2 | 1 | 0 | 0 | 0 | 0 |
| ∑ | 6 | 5 | 2 | 1 | 0 | 0 | 0 | 0 |
| % ∑ | 85.7 | 71.4 | 28.6 | 14.3 | 0 | 0 | 0 | 0 |
| % missing | 42.9 | 0 | 0 | 0 | 0 | 0 | 0 | 0 |
| % deformed | 42.9 | 71.4 | 28.6 | 14.3 | 0 | 0 | 0 | 0 |

| | Only Lr | Headless | Dorsal closure | Small abd | Abd i-o |
|---|---|---|---|---|---|
| n | 4 | 0 | 2 | 0 | 0 |
| % | 57.1 | 0 | 28.6 | 0 | 0 |

**Cuticle defects after injection of *Tc-importin α1*a dsRNA (0.3 µg/µl) in *SB* female pupae which were crossed to *SB* males.**

| ID | impα1a SBxSB |
|---|---|
| dsRNA[ µg/µl] | 0.3 |
| n | 18 |
| wt | 2 |
| pt | 16 |
| % affected | 88.9 |

| | Lr | Head b.p. | At | Gnath. app. | T1-3 | A1-8 | Urogomphi | Pygopods |
|---|---|---|---|---|---|---|---|---|
| missing | 0 | 0 | 0 | 1 | 0 | 8 | 8 | 8 |
| deformed | 2 | 13 | 0 | 1 | 4 | 5 | 0 | 0 |
| ∑ | 2 | 13 | 0 | 2 | 4 | 13 | 8 | 8 |
| % ∑ | 12.5 | 81.3 | 0 | 12.5 | 25.0 | 81.3 | 50.0 | 50.0 |
| % missing | 0 | 0 | 0 | 6.3 | 0 | 50.0 | 50.0 | 50.0 |
| % deformed | 12.5 | 81.3 | 0 | 6.3 | 25.0 | 31.3 | 0 | 0 |

| | Onyl Lr | Headless | Dorsal closure | Small abd | Abd i-o |
|---|---|---|---|---|---|
| n | 0 | 0 | 1 | 0 | 8 |
| % | 0 | 0 | 6.3 | 0 | 50.0 |

**Cuticle defects after injection of *Tc-importin α1*a dsRNA (0.3 µg/µl) in *black* female pupae which were crossed to *black* males.**

| | |
|---|---|
| **ID** | impα1a BlxBl |
| **dsRNA[µg/µl]** | 0.3 |
| **n** | 18 |
| **wt** | 1 |
| **pt** | 17 |
| **% affected** | 94.4 |

| | **Lr** | **Head b.p.** | **At** | **Gnath. app.** | **T1-3** | **A1-8** | **Urogomphi** | **Pygopods** |
|---|---|---|---|---|---|---|---|---|
| **missing** | 7 | 0 | 1 | 0 | 0 | 0 | 0 | 0 |
| **deformed** | 7 | 14 | 1 | 0 | 3 | 3 | 0 | 0 |
| **∑** | 14 | 14 | 2 | 0 | 3 | 3 | 0 | 0 |
| **% ∑** | 82.4 | 82.4 | 11.8 | 0 | 17.6 | 17.6 | 0 | 0 |
| **% missing** | 41.2 | 0 | 5.9 | 0 | 0 | 0 | 0 | 0 |
| **% deformed** | 41.2 | 82.4 | 5.9 | 0 | 17.6 | 17.6 | 0 | 0 |

| | **Only Lr** | **Headless** | **Dorsal closure** | **Small abd** | **Abd i-o** |
|---|---|---|---|---|---|
| **n** | 14 | 0 | 2 | 0 | 0 |
| **%** | 82.4 | 0 | 11.8 | 0 | 0 |

**Cuticle defects after injection of *Tc-importin α1*a dsRNA (3 µg/µl) in *black* female pupae which were crossed to *black* males.**

| | |
|---|---|
| **ID** | impα1a BlxBl |
| **dsRNA[µg/µl]** | 3 |
| **n** | 20 |
| **wt** | 0 |
| **pt** | 20 |
| **% affected** | 100 |

| | **Lr** | **Head b.p.** | **At** | **Gnath. app.** | **T1-3** | **A1-8** | **Urogomphi** | **Pygopods** |
|---|---|---|---|---|---|---|---|---|
| **missing** | 8 | 0 | 2 | 4 | 0 | 2 | 2 | 2 |
| **deformed** | 4 | 19 | 4 | 3 | 1 | 1 | 0 | 0 |
| **∑** | 12 | 19 | 6 | 7 | 1 | 3 | 2 | 2 |
| **% ∑** | 60.0 | 95.0 | 30.0 | 35.0 | 5.0 | 15.0 | 10.0 | 10.0 |
| **% missing** | 40.0 | 0 | 10.0 | 20.0 | 0 | 10.0 | 10.0 | 10.0 |
| **% deformed** | 20.0 | 95.0 | 20.0 | 15.0 | 5.0 | 5.0 | 0 | 0 |

| | **Only Lr** | **Headless** | **Dorsal closure** | **Small abd** | **Abd i-o** |
|---|---|---|---|---|---|
| **n** | 6 | 0 | 3 | 0 | 1 |
| **%** | 30.0 | 0 | 15.0 | 0 | 5.0 |

## Cuticle defects after injection of *Tc-importin α1*a dsRNA (1 µg/µl) in *SB* female pupae which were crossed to *black* males.

| ID | impα1a SBxBl |
|---|---|
| dsRNA[µg/µl] | 1 |
| n | 25 |
| wt | 2 |
| pt | 23 |
| % affected | 92.0 |

| | Lr | Head b.p. | At | Gnath. app. | T1-3 | A1-8 | Urogomphi | Pygopods |
|---|---|---|---|---|---|---|---|---|
| missing | 2 | 2 | 4 | 4 | 2 | 13 | 13 | 13 |
| deformed | 4 | 13 | 2 | 2 | 9 | 1 | 0 | 0 |
| ∑ | 6 | 15 | 6 | 6 | 11 | 14 | 13 | 13 |
| % ∑ | 26.1 | 65.2 | 26.1 | 26.1 | 47.8 | 60.9 | 56.5 | 56.5 |
| % missing | 8.7 | 8.7 | 17.4 | 17.4 | 8.7 | 56.5 | 56.5 | 56.5 |
| % deformed | 17.4 | 56.5 | 8.7 | 8.7 | 39.1 | 4.3 | 0 | 0 |

| | Only Lr | Headless | Dorsal closure | Small abd | Abd i-o |
|---|---|---|---|---|---|
| n | 1 | 2 | 4 | 3 | 10 |
| % | 4.3 | 8.7 | 17.4 | 13.0 | 43.5 |

## Cuticle defects after injection of *Tc-importin α1*a dsRNA (1 µg/µl) in *black* female pupae which were crossed to *SB* males.

| ID | impα1a BlxSB |
|---|---|
| dsRNA[µg/µl] | 1 |
| n | 27 |
| wt | 3 |
| pt | 24 |
| % affected | 88.9 |

| | Lr | Head b.p. | At | Gnath. app. | T1-3 | A1-8 | Urogomphi | Pygopods |
|---|---|---|---|---|---|---|---|---|
| missing | 12 | 1 | 3 | 1 | 0 | 2 | 3 | 3 |
| deformed | 7 | 20 | 2 | 3 | 2 | 2 | 0 | 0 |
| ∑ | 19 | 21 | 5 | 4 | 2 | 4 | 3 | 3 |
| % ∑ | 79.2 | 87.5 | 20.8 | 16.7 | 8.3 | 16.7 | 12.5 | 12.5 |
| % missing | 50.0 | 4.2 | 12.5 | 4.2 | 0 | 8.3 | 12.5 | 12.5 |
| % deformed | 29.2 | 83.3 | 8.3 | 12.5 | 8.3 | 8.3 | 0 | 0 |

| | Only Lr | Headless | Dorsal closure | Small abd | Abd i-o |
|---|---|---|---|---|---|
| n | 12 | 0 | 2 | 1 | 0 |
| % | 50.0 | 0 | 8.3 | 4.2 | 0 |

**F) Tables summarizing the defects found in the other RNAi experiments**

**Quantification of cuticle phenotypes after *Tc-dll* RNAi in *black* female pupae crossed to *black* males.**

| ID | *Tc-dll* BlxBl |
|---|---|
| dsRNA[µg/µl] | 1 |
| n | 50 |

| Phenotype | Level 4 | Level 3 | Level 2 | Level 1 |
|---|---|---|---|---|
| ∑ | 6 | 16 | 21 | 7 |
| % | 12 | 32 | 42 | 14 |

**Quantification of cuticle phenotypes after *Tc-dll* RNAi in *SB* female pupae crossed to *SB* males.**

| ID | *Tc-dll* SBxSB |
|---|---|
| dsRNA[µg/µl] | 1 |
| n | 50 |

| Phenotype | Level 4 | Level 3 | Level 2 | Level 1 |
|---|---|---|---|---|
| ∑ | 0 | 14 | 33 | 3 |
| % | 0 | 28 | 66 | 6 |

**Quantification of cuticle phenotypes after *Tc-gt* RNAi in *SB* female pupae crossed to *SB* males.**

| ID | *Tc-gt* SBxSB |
|---|---|
| dsRNA[µg/µl] | 1 |
| n | 50 |

| Del. segm. n | 0 | 0.5 | 1 | 1.5 | 2 | 2.5 | 3 | 3.5 | 4 | 4.5 | 5 | 5.5 | 6 | 6.5 | 7 |
|---|---|---|---|---|---|---|---|---|---|---|---|---|---|---|---|
| ∑ | 1 | 4 | 3 | 9 | 7 | 11 | 5 | 4 | 3 | 2 | 0 | 0 | 1 | 0 | 0 |
| % | 2 | 8 | 6 | 18 | 14 | 22 | 10 | 8 | 6 | 4 | 0 | 0 | 2 | 0 | 0 |

**Quantification of cuticle phenotypes after *Tc-gt* RNAi in *black* female pupae crossed to *black* males.**

| ID | *Tc-gt* BlxBl |
|---|---|
| dsRNA[µg/µl] | 1 |
| n | 50 |

| Del. segm. n | 0 | 0.5 | 1 | 1.5 | 2 | 2.5 | 3 | 3.5 | 4 | 4.5 | 5 | 5.5 | 6 | 6.5 | 7 |
|---|---|---|---|---|---|---|---|---|---|---|---|---|---|---|---|
| ∑ | 0 | 1 | 6 | 6 | 11 | 10 | 8 | 7 | 1 | 0 | 0 | 0 | 0 | 0 | 0 |
| % | 0 | 2 | 12 | 12 | 22 | 20 | 16 | 14 | 2 | 0 | 0 | 0 | 0 | 0 | 0 |

**Quantification of pupal phenotypes after *Tc-wg* lRNAi in *SB* females.**

| ID | *Tc-wg* SB |
|---|---|
| dsRNA[µg/µl] | 1 |
| n | 10 |

| Phenotype | Level 4 | Level 3 | Level 2 | Level 1 |
|---|---|---|---|---|
| Mx ø [in] | 0,16-0,21 | 0,22-0,27 | 0,28-0,33 | >0,33 |
| ∑ | 5 | 1 | 1 | 3 |
| % | 45.5 | 9.1 | 9.1 | 27.3 |

## Quantification of pupal phenotypes after *Tc-wg* lRNAi in *black* females.

| ID | *Tc-wg* Bl |
|---|---|
| dsRNA[µg/µl] | 1 |
| n | 12 |

| Phenotype | Level 4 | Level 3 | Level 2 | Level 1 |
|---|---|---|---|---|
| Mx ø [in] | 0,16-0,21 | 0,22-0,27 | 0,28-0,33 | >0,33 |
| ∑ | 3 | 6 | 2 | 1 |
| % | 25 | 50 | 16.7 | 8.3 |

## Quantification of pupal phenotypes after *Tc-six3* lRNAi in *black* females.

| ID | *Tc-six3* Bl |
|---|---|
| dsRNA[µg/µl] | 0.5 |
| n | 10 |

| Phenotype | Level 4 | Level 3 | Level 2 | Level 1 |
|---|---|---|---|---|
| Eyefield [in²] | <0.16 | 0.17-0.23 | 0.24-0.30 | >0.31 |
| ∑ | 3 | 4 | 1 | 2 |
| % | 30 | 40 | 10 | 20 |

## Quantification of pupal phenotypes after *Tc-six3* lRNAi in *SB* females.

| ID | *Tc-six3* SB |
|---|---|
| dsRNA[µg/µl] | 0.5 |
| n | 11 |

| Phenotype | Level 4 | Level 3 | Level 2 | Level 1 |
|---|---|---|---|---|
| Eyefield [in²] | <0.16 | 0.17-0.23 | 0.24-0.30 | >0.31 |
| ∑ | 2 | 8 | 1 | 0 |
| % | 18.2 | 72.7 | 9.1 | 0 |

## G) Measurements of maxillary palpus diameter and eyefield

### *SB* wild-type pupae

| ID | wt *SB* |
|---|---|
| n | 10 |

| | Mx ø [in] | Eyefield [in²] |
|---|---|---|
| 1 | 0.35 | 0.41 |
| 2 | 0.35 | 0.41 |
| 3 | 0.35 | 0.43 |
| 4 | 0.33 | 0.41 |
| 5 | 0.36 | 0.4 |
| 6 | 0.35 | |
| 7 | 0.36 | |
| 8 | 0.35 | |
| 9 | 0.34 | |
| 10 | 0.36 | |

### *black* wild-type pupae.

| ID | wt *black* |
|---|---|
| n | 10 |

| | Mx ø [in] | Eyefield [in²] |
|---|---|---|
| 1 | 0.35 | 0.46 |
| 2 | 0.35 | 0.45 |
| 3 | 0.35 | 0.45 |
| 4 | 0.35 | 0.46 |
| 5 | 0.37 | 0.41 |
| 6 | 0.35 | |
| 7 | 0.37 | |
| 8 | 0.35 | |
| 9 | 0.35 | |
| 10 | 0.36 | |

## H) Alignment of *Tc*-I*mportin α1* amino acid sequence in different Tribolium strains and metazoans

>*S. cerevisae* SRP1
MDNGTDSSTSKFVPEYRRTNFKNKGRFSADELRRRRDTQQVELRKAKRDEALAKRRNFIPPTDGADSDEEDESSVSADQQFYSQLQQELPQM
TQQLNSDDMQEQLSATVKFRQILSREHRPPIDVVIQA-
GVVPRLVEFMRENQPEMLQLEAAWALTNIASGTSAQTKVVVDADAVPLFIQLLYTGSVEVKEQAIWALGNVAGDSTDYRDYVLQCNAMEPIL
GLFN-
SNKPSLIRTATWTLSNLCRGKKPQPDWSVVSQALPTLAKLIYSMDTETLVDACWAISYLSDGPQEAIQAVIDVRIPKRLVELLSHESTLVQTPALR
AVGNIVTGNDLQTQVVINAGVLPALRLLLSSPKENIKKEACWTISNITAGNTEQIQAVIDANLIPPLVKLLEVAEYKTKKEACWAISNASSGGLQR
PDIIRYLVSQGCIKPLCDLLEIADNRIIEVTLDALENILKMGEADKEARGLNINENADFIEKAGGMEKIFNCQQNENDKIYEKAYKIIETYFGEEEDA
VDETMAPQNAGNTFGFGSNVN-----QQFNFN_

>*T. castaneum SB*
-------MS-GSAHKHR---YKNAA-LDSVELRRRRREEEGLQLRKQKREQQLNKRRNVN--VNQLAEDNDHLQVDSEILSPVS--
PMITPEMVQALYSPDVEQQISATQKFRQLLSYEPNPPIDEVVQT-
GIIPRFVEFLQNSNNCNLQFEAAWALTNVASGTSQQTRMVIEAGAVPIFIRLLSSQYEDVQEQAVWALGNIAGDSPECRDHVLDSGILVPLLQL
LSKSTRLSMTRNAVWALSNLCRGKNPPPDFAKVSPALPVLARLLFHSDPDVLSDTCWALSYLSDGPNEKIQAVIDAGVCRKLVELLMHQQPNV
VSAALRAVGNIVTGDDVQTQVILNCSALHCLHHLLSSSKESVRKEACWTISNITAGNRLQIQAVIDANIFPVLIEILSKAEFKTRKEAAWAITNATS
GGT--PDQIRYLVNQACIGPLCDLLTVMDAKIVQVALNGLENILRLGEQDAKN-
HSGTNPYAVLIEQCYGLDKIEFLQSHVNMEIYQKAFDIIEHFFGTEEEDTTVAPSVNPDQQQYHFSSDQS--VPMGGFHF_-

>*T. castaneum Black*
-------MS-GSAHKHR---YKNAA-LDSVELRRRRREEEGLQLRKQKREQQLNKRRNVN--VNQLAEDNDHLQVDSEILSPVS--
PMITPEMVQALYSPDVEQQISATQKFRQLLSYEPNPPIDEVVQT-
GIIPRFVEFLQNSNNCSLQFEAAWALTNVASGTSQQTRMVIEAGAVPIFIRLLSSQYEDVQEQAVWALGNIAGDSPECRDHVLDSGILVPLLQLL
SKSTRLSMTRNAVWALSNLCRGKNPPPDFAKVSPALPVLARLLFHSDPDVLSDTCWALSYLSDGPNEKIQAVIDAGVCRKLVELLMHQQPNVV
SAALRAVGNIVTGDDVQTQVILNCSALHCLHHLLSSSKESVRKEACWTISNITAGNRQQIQAVIDANIFPVLIEILSKAEFKTRKEAAWAITNATS
GGT--PDQIRYLVNQACIGPLCDLLTVMDAKIVQVALNGLENILRLGEQDAKN-
HSGTNPYAVLIEQCYGLDKIEFLQSHVNMEIYQKAFDIIEHFFGTEEEDTTVAPSVNPDQQQYHFSSDQS--VPMGGFHF_-

>*T. castaneum GA2*
-------MS-GSAHKHR---YKNAA-LDSVELRRRRREEEGLQLRKQKREQQLNKRRNVN--VNQLAEDNDHLQVDSEILSPVS--
PMITPEMVQALYSPDVEQQISATQKFRQLLSYEPNPPIDEVVQT-
GIIPRFVEFLQNSNNCSLQFEAAWALTNVASGTSQQTRMVIEAGAVPIFIRLLSSQYEDVQEQAVWALGNIAGDSPECRDHVLDSGILVPLLQLL
SKSTRLSMTRNAVWALSNLCRGKNPPPDFAKVSPALPVLARLLFHSDPDVLSDTCWALSYLSDGPNEKIQAVIDAGVCRKLVELLMHQQPNVV
SAALRAVGNIVTGDDVQTQVILNCSALHCLHHLLSSSKESVRKEACWTISNITAGNRQQIQAVIDANIFPVLIEILSKAEFKTRKEAAWAITNATS
GGT--PDQIRYLVNQACIGPLCDLLTVMDAKIVQVALNGLENILRLGEQDAKN-
HSGTNPYAVLIEQCYGLDKIEFLQSHVNMEIYQKAFDIIEHFFGTEEEDTTVAPSVNPDQQQYHFSSDQS--VPMGGFHF_-

>*M .musculus*
----METMASPGKDNYRMKSYKNNA-LNPEEMRRRRREEEGIQLRKQKREQQLFKRRNVE--
LINEEAAMFDSLLMDSYVSSTTGESVITREMVEMLFSDDSDLQLATTQKFRKLLSKEPSPPIDEVINTPGVVDRFVEFLKRNENCTLQFEAAWAL
TNIASGTSQQTKIVIEAGAVPIFIELLNSDFEDVQEQAVWALGNIAGDSSLCRDYVLNCSILNPLLTLLTKSTRLTMTRNAVWALSNLCRGKNPPP
EFAKVSPCLPVLSRLLFSSDSDLLADACWALSYLSDGPNEKIQAVIDSGVCRRLVELLMHNDYKVASPALRAVGNIVTGDDIQTQVILNCSALPCL
LHLLSSSKESIRKEACWTISNITAGNRAQIQAVIDANIFPVLIEILQKAEFRTRKEAAWAITNATSGGT--
PEQIRYLVSLGCIKPLCDLLTVMDSKIVQVALNGLENILRLGEQESKRSGSGVNPYCGLIEEAYGLDKIEFLQSHENQEIYQKAFDLIEHYFGVEDD
DSSLAPQVDETQQQFIFQQPEA---PMEGFQL_-

>*D. melanogaster*
-----------------------------------------------------------------------------------------VINEEMIQMLFSGRENEQLEATQKFRKLLSRDPNPPIEEVIQK-
GIVPQFVTFLRNSANATLQFEAAWTLTNIASGTSQQTKVVIEAGAVPIFIDLLSSPHDDVQEQAVWALGNIAGDSPMCRDHLLGSGILEPLLHV
LSNSDRITMIRNAVWTLSNLCRGKSPPADFAKISHGLPILARLLKYTDADVLSDTCWAIGYLSDGPNDKIQAVIDAGVCRRLVELLLHPQQNVST
AALRAVGNIVTGDDQQTQVILGYNALTCISHLLHSTAETIKKESCWTISNIAAGNREQIQALINANIFPQLMVIMQTAEFKTRKEAAWAITNATS
SGT--HEQIHYLVQVGCVPPMCDFLTVVDSDIVQVALNALENILKAGEKFQTR----
PNPYAITIEECGGLDKIEYLQAHENRDIYHKSFYIIEQYFGNEEEDSRVAPVAG--TQQFEFD-PDN--MPSTGFNF_-

>*A. mellifera*
-------MSAATTHKYR---YKNVG-LDSQELRRRRREEEGVQLRKQKREQQLSKRRNVP--NMVADDDNVTGNES-
AYPAPQSKTSIIVLEMVQELYSPNPEAQLAATQKFRKMLSREPNPPIDEVVKT-
GIVPKFVEFLENNANCTLQFEAAWALTNIASGTSQQTRVVVDAGAVPIFISLLGSEYEDVQEQAVWALGNIAGDSPECRDHVLDRGILTPLLQLL
SKATRLSMTRNAVWALSNLCRGKTPPPEFTKVAPCLPVLAHFLNHTDSDVLADACWALSYLSDGPNEKIQAVIDAGVCRRLVELLMHQQENV
VSAALRAVGNIVTGDDVQTQIVLNCSALHCLYHLLNSSQESIRKEACWTISNITAGNPQQIQAVIDAGIFPVLIDILAKAEFKIRKEAAWAITNATS

GGA--PEQIRYIVVEGCIPPLCNLLTVMDPKIVQVALSGLENILRVGEQDAAS-
HNGINHYAVLIEECFGLNKIEFLQSHQNVEIYQKAFDIIERYFGSEEEDTRVVPTIDAQGQQFQFRAPDSSQLPVQGFEF_-

>*N. vitripennis*
-------MSSGPAHKYR---YKNVG-LDSQELRRRREEEGVQLRKQKREQQLSKRRNVP--
NIVTDDDGVSITEDYTVPTPIMQAPGITQDMVEALYSQDVNEQLTATQKFRKLLSKEPNPPIDEVIQT-
GIVPRFVEFLKNDTNCTLQFEAAWALTNIASGTSLQTRMVVDAGAVPMFIALLSSEYEDVQEQAVWALGNIAGDSPACRDHVLSSGILPPLLVL
LSKPARLTMTRNAVWALSNLCRGKEPAPDFQKVAPCLPVLAYLLNHQDADVLADACWALSYISDGPNEKIQAVIDAGVCRRLVELLMHEQHN
VTSAALRAVGNIVTGDDMQTQVVLNCSALTCLLHLLGQSRESIRKEACWTVSNITAGNPQQIQAVIDAKIFPVIIDILGKAEFKTRKEAAWAITN
ATSGGT--ADQIRYLADQNCIPPLCELLTVMDATIIQVALNGLENILRLGEQDAVH-
HNGVNPYCVLIEQCFGLDKIEFLQSHQNLEIYQKAFDIIERYFGSEEEDSKIAPSVDSQGQQFQFPAPDSSQLPVHSFQF_-

## I) Phenotype of pig19 injected females mated to black males

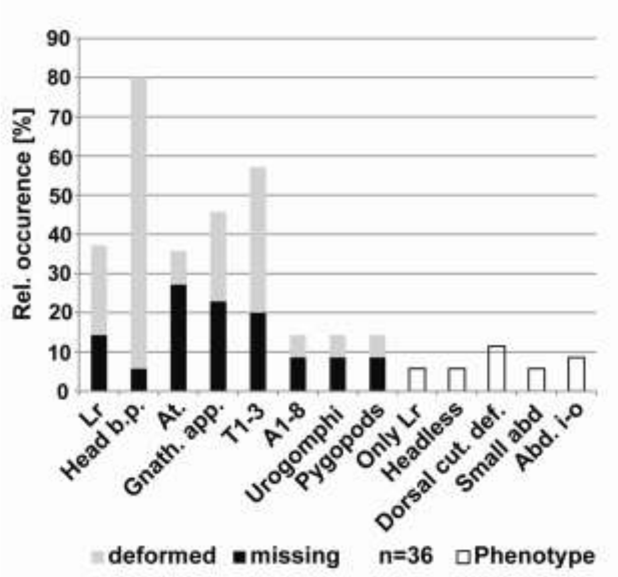


The phenotype of the offspring is an intermediate between black and SB in that “only labrum” phenotypes do occur (6%) but more structures are affected than in black (compare to Fig. A and D)

## J) Effect of *Tc*-I*mportin α1*RNAi on fertility of different strains

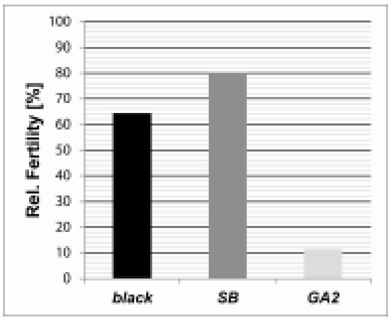


*Tc-dll* (control) and *Tc-importin α1*a dsRNA (both 1 µg/µl) were injected into female pupae of *Black*, *SB* and *Georgia-2* and mated to untreated males of the same strain. The eggs laid per female were documented and the *Tc-importin α 1*a RNAi results were normalized to the control RNAi. *Tc-importin α1*a RNAi in the *Black* and *SB* background resulted in a reduction of the egglay of about 20% (*SB*) to 35% (*black*). The egglay of *GA2* was reduced by about 90%.

**K) Upregulation of maternal *Tc-importin α2* after *Tc-importin α1* RNAi in the *black* strain**

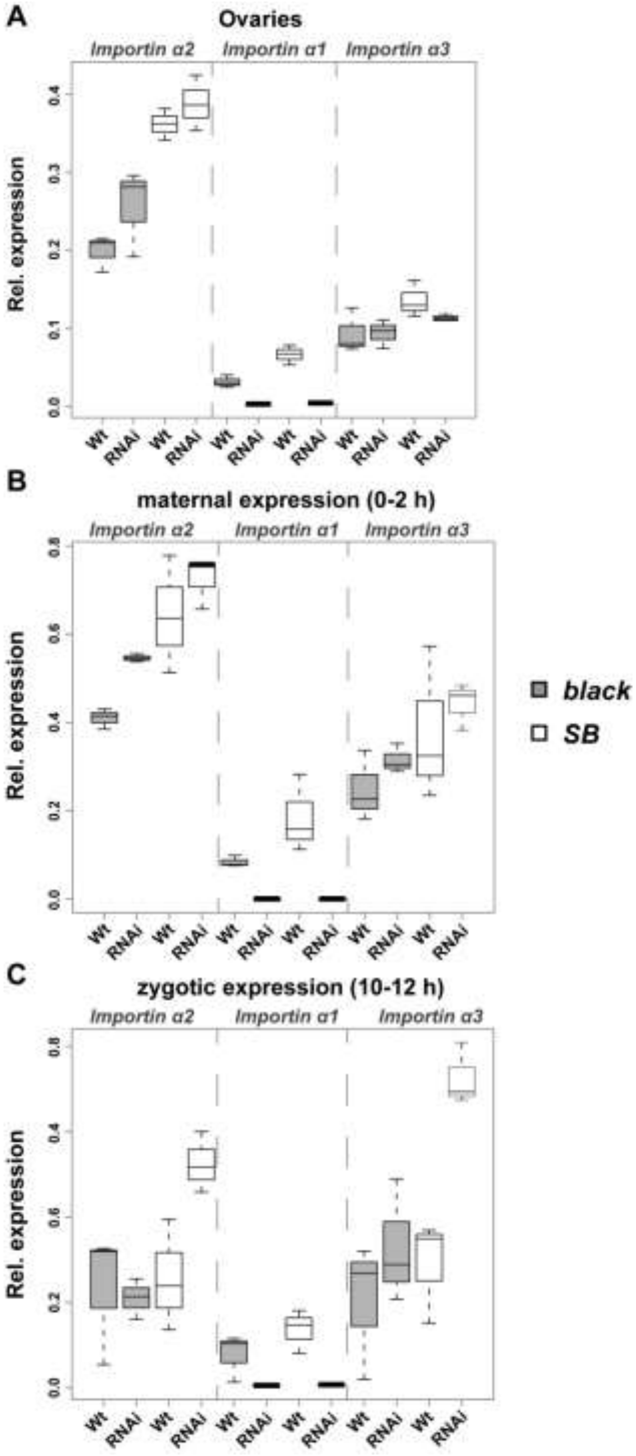


The level of expression was determined by qPCR in ovaries (A), freshly laid eggs (0-2h; maternal message; B) and 10-12 h embryos (zygotic expression; C) in wild-type and in *Tc-importin α1* knockdown animals. Results from the black strain are depicted with grey boxes, *SB* expression levels are shown as open boxes. wt and RNAi values are shown side by side, respectively. B) In *Tc-importin α1* knockdown embryos, the maternal load of *Tc-importin α2* is strongly elevated in *black* (compare first with second column) but less so in *SB*. C) Zygotic expression of *Tc-importin α2* and *3* are elevated in *SB* but less so in *black* (compare fourth with third and 12th with 11th column). Note that in contrast to figure 8, the values of the two strains were not normalized to each other.
